# Identification of the neurotransmitter profile of AmFoxP expressing neurons in the honeybee brain using double-label in situ hybridization

**DOI:** 10.1186/s12868-018-0469-1

**Published:** 2018-11-06

**Authors:** Adriana Schatton, Julia Agoro, Janis Mardink, Gérard Leboulle, Constance Scharff

**Affiliations:** 1 Department of Animal Behavior, Freie Universität Berlin, Takustraße 6, 14195 Berlin, Germany; 2 Department of Neurobiology, Freie Universität Berlin, Königin-Luise-Straße 28-30, 14195 Berlin, Germany

**Keywords:** FoxP, FoxP1, Honeybee, Acetylcholine, Glutamate, GABA, Monoamine, Songbird, Deep homology, In situ hybridization

## Abstract

**Background:**

FoxP transcription factors play crucial roles for the development and function of vertebrate brains. In humans the neurally expressed FOXPs, FOXP1, FOXP2, and FOXP4 are implicated in cognition, including language. Neural FoxP expression is specific to particular brain regions but FoxP1, FoxP2 and FoxP4 are not limited to a particular neuron or neurotransmitter type. Motor- or sensory activity can regulate FoxP2 expression, e.g. in the striatal nucleus Area X of songbirds and in the auditory thalamus of mice. The DNA-binding domain of FoxP proteins is highly conserved within metazoa, raising the possibility that cellular functions were preserved across deep evolutionary time. We have previously shown in bee brains that FoxP is expressed in eleven specific neuron populations, seven tightly packed clusters and four loosely arranged groups.

**Results:**

The present study examined the co-expression of honeybee FoxP (AmFoxP) with markers for glutamatergic, GABAergic, cholinergic and monoaminergic transmission. We found that AmFoxP could co-occur with any one of those markers. Interestingly, AmFoxP clusters and AmFoxP groups differed with respect to homogeneity of marker co-expression; within a cluster, all neurons co-expressed the same neurotransmitter marker, within a group co-expression varied. We also assessed qualitatively whether age or housing conditions providing different sensory and motor experiences affected the AmFoxP neuron populations, but found no differences.

**Conclusions:**

Based on the neurotransmitter homogeneity we conclude that AmFoxP neurons within the clusters might have a common projection and function whereas the AmFoxP groups are more diverse and could be further sub-divided. The obtained information about the neurotransmitters co-expressed in the AmFoxP neuron populations facilitated the search of similar neurons described in the literature. These comparisons revealed e.g. a possible function of AmFoxP neurons in the central complex. Our findings provide opportunities to focus future functional studies on invertebrate FoxP expressing neurons. In a broader context, our data will contribute to the ongoing efforts to discern in which cases relationships between molecular and phenotypic signatures are linked evolutionary.

## Background

Transcription factors of the FOXP family are intensively investigated because of their role in human disease [1]. Human patients with mutations in FOXP1, FOXP2 or FOXP4 show several cognitive deficits such as autistic features, mental retardation and language impairments [2–6]. A point mutation in FOXP2, initially discovered in one large family, impairs speech perception and production while non-language-related behaviors are less affected [7, 8]. More than 30 genetic alterations of FOXP2 in other individuals also affect language in similar ways, and are additionally associated with autistic spectrum disorders [5, 9–11]. In the following text we adopted the nomenclature proposed by Kaestner et al. [12], e.g. human gene *FOXP* and protein FOXP, mouse (*Foxp2/*Foxp2) and all other species (*FoxP2*/FoxP2).

FoxP gene expression in the CNS is overall similar in all vertebrates analyzed and includes regions involved in sensory-motor and multimodal integration [13–23].

Invertebrate genomes harbor only one *FoxP* gene locus that contains a highly conserved DNA-binding domain [24, 25]. In sponges FoxP is upregulated in vitro during the formation of cell aggregates [26]. In *Drosophila*, FoxP (dFoxP) has been implicated in decision-making, locomotion and motor learning [27–30]. In honeybees, FoxP (AmFoxP) is expressed in the cortical areas of all major neuropils, either in dense neuron clusters or more widely dispersed neuron groups [25, 31, 32]. The *FoxP* gene is most similar to vertebrate *FoxP1*, based on particular alternative splice forms in bilaterians [25, 33].

We previously described eleven AmFoxP expressing neuron populations in particular locations of the honeybee brain [25]. We chose to study the role of FoxP in the honeybee *Apis mellifera* because it is a well-established animal model to study insect cognition [34, 35] such as memory formation [36], navigation [37], symbolic communication [38–41] or conceptual learning [42–44].

For one of the described AmFoxP clusters, the posterior cluster medioventral to the lobula (mvLO-p), we proposed, based on location and connectivity, a functional role in visual processing [25, 31], consistent with FoxP expressing neurons in the mammalian visual thalamus [45, 46]. Here we further characterize the AmFoxP neuron populations in the honeybee brain in terms of their neurotransmitters to better compare them with neurons of known function in the insect CNS. Moreover, we also studied whether environmental stimuli affected *AmFoxP* expression in honeybees, because FoxP2 can be regulated by activity in some vertebrate neurons, e.g. by light, mtor behavior and/or sound [13, 47–49].

In honeybees, neurotransmitter systems were described by various means, including the detection of the neurotransmitters themselves [50–55], of the enzymes implicated in their metabolism [56–58], of molecules that transport the neurotransmitters into cellular compartments [59] and of specific receptors [60–67].

We concentrated on the most relevant neurotransmitters by means of their markers: the honeybee (Am) vesicular transporters for acetylcholine (AmVAchT), glutamate (AmVGluT) and monoamines (AmVMAT) as well as glutamate decarboxylase (AmGad). These markers were chosen because they are exclusively associated with presynaptic release (transporters) or synthesis (Gad) of the respective neurotransmitter. To our knowledge, VAchT, VGluT and Gad have not been reported to be expressed endogenously in glia cells. *Drosophila* VMAT is also expressed in glia cells, however restricted to serotonin and histamine containing neurons in a relatively thin layer between the retina and the optic lobe [68] where we did not detect AmFoxP. Acetylcholine (Ach) is the main excitatory neurotransmitter in insects, in contrast to vertebrates that predominantly use glutamate for excitatory transmission [69, 70]. Excitatory currents induced by Ach are characterized [71, 72]. Glutamatergic signaling in honeybees is important for particular memory mechanisms [63, 73, 74]. Inhibitory currents induced by Glutamate (Glu) through glutamate chloride channels (GluCl) have also been described [72, 75]. The detection of receptors homologous to their vertebrate counterparts (AMPA/kainate, NMDA) suggests that Glu also acts as an excitatory neurotransmitter in the CNS [61]. Another major inhibitory neurotransmitter in the bilaterian brain is GABA [72, 76, 77]. GluCl and GABA receptors are both involved in honeybee long term memory [78, 79]. AmVMAT transports the biogenic amines octopamin, dopamine, histamine and serotonin [68, 80] and thus serves as a marker for modulatory neurons. In insects, modulatory neurons are crucial for different learning processes [81–83], involved in the division of labour [84, 85] and mediate the biological state of the organism like aggression, sleep or hunger [86–88]. For the quantitative PCR analysis of neurotrasmitters expressed in the Kenyon cells of the mushroom bodies, we used, beside AmVachT, AmVGluT and AmVMAT, also AmVIAAT (the honeybee vesicular transporter for inhibitory amino acids). AmVIAAT is a presynaptic marker for glycinergic and GABAergic neurons.

Although insect and vertebrate brains demonstrate fundamental differences in terms of development, anatomy, structure, design and complexity, they also have many principle features in common [89]. Some of those might be convergent whereas others, like the regulatory networks and molecular architecture of specific neurons could date back to their last common bilaterian ancestor and are therefore ‘deeply homologous’ [90]. In the framework of deep homology between insect and vertebrate brains [90, 91], we are interested in potentially conserved features of FoxP expressing neurons with implications for similarities and differences in behavior.

## Methods

### Animals

All honeybees of the species *A. mellifera* were collected between July and September from apiaries located at the department of Neurobiology, FU Berlin (Königin-Luise-Str. 1-3, 14195 Berlin). Access was generously provided by Professors Randolf Menzel and Dorothea Eisenhardt.

### Sampling method

To address whether differences in experience or life history affected *AmFoxP* expression we created four conditions that varied with respect to sensory experience and opportunity for motor activity. From each condition we sampled bees of different ages. In condition 1 bees were kept in an observation hive with two frames (W × D × H: 53.5 × 18 × 65 cm), in the presence of the queen and siblings (~ 4000 individuals), a honeycomb and the possibility to fly and forage outside of the hive (‘unmanipulated’ group). In order to sample bees of the unmanipulated group at specific ages, they were marked with acrylic color on their thorax within 16 h after emergence. In the other three conditions one or more variables were altered (Table 1): In condition 2 (‘honeycomb’ group) ~ 100 individuals were kept in a caged honeycomb frame (22 cm × 37 cm × 5 cm). In condition 3 (‘mini-cage’ group) ~ 30 bees were kept separately within the hive, enclosed in a small cage (10 cm × 3 cm × 10 cm) without an own honeycomb. In condition 4 (‘incubator’ group) ~ 30 bees were kept in an empty cage (13 cm × 20 cm × 8 cm) within an incubator at 29 °C in constant darkness and supplied with 30% sucrose.

**Table 1:** Treatment and sample size of the four experimental groups

Condition/group	Sisters	Queen	Honeycomb	Foraging/light	Number and ages of individuals sampled
‘Unmanipulated’	+	+	+	+	3 (age: 1 × 1 days, 1 × 15 days, 1 × 50 days)
‘Honeycomb’	+	+	+	–	4 (age: 1 × 4 days, 1 × 11 days, 1 × 15 days, 1 × 19 days)
‘Mini-cage’	+	+	–	–	3 (age: 2 × 23 days, 1 × 26 days)
‘Incubator’	+	–	–	–	5 (age: 2 × 15 days, 1 × 27 days, 1 × 31 days, 1 × 40 days)

The manipulated features are indicated by symbols. The column on the right lists the sample size. The detailed distribution of age (*d* = days after emergence) within the group is given in brackets

### Preparation of in situ hybridization probes

Table 2 provides an overview of the primers used to prepare in situ hybridization (*ISH*) probes. In the following text, nucleotide sequences (*ISH*-probes, primers) are written in *italics* and ‘*Am*’ as a prefix refers to ‘*Apis* *mellifera*’. The *AmFoxP* probe-containing pGemT-easy plasmid was kindly provided by Prof. Taketoshi Kiya (Kanazawa University, Japan). The same probe was used previously [25] and does not discriminate between the two the Forkhead domain—affecting isoforms [25]. The *AmGad*-probe was produced according to Kiya and Kubo [58]. Honeybee sequences were identified with BLASTN 2.7.0+ using the ‘blastn’ algorithm [92] (RRID: 196SCR_001010) and phylogenetic analysis (clustal omega, EMBL-EBI, RRID:SCR_001591) by comparing to *Drosophila* and mammalian orthologous genes available in sequence databases. Primers (mwg Eurofins Genomics, Ebersberg, Germany) were identified with ‘primer3’ software [93] (RRID:SCR_002285) and used to PCR-amplify sequences between 500 and 1000 nt to serve as *ISH* templates. Template cDNA was prepared as follows: freshly dissected honeybee brains were homogenized with a pellet pestle (Sigma-Aldrich, Germany) in 400 µl TriZol^®^. After adding 80 µl of chloroform the solution was vortexed and centrifuged (15 min at 12,000 *g* at 4 °C), the RNA-containing upper layer was collected and purified on columns (‘RNeasy MinElute Cleanup’ by Qiagen, Hilden, Germany) according to the manufacturer’s protocol. Residual DNA was restricted with TurboDNase^®^ (Thermo Fisher, Braunschweig, Germany). RNA concentration was measured with a NanoDrop 1000 Spectrophotometer. 200 ng of total RNA were transcribed into cDNA by using oligo-dT-primers and SuperScript^®^ III Reverse Transcriptase (Thermo Fisher, Braunschweig, Germany).

**Table 2:** RNA probe names used for in situ hybridization

Probe name	Primers 5′–3′	Ref. seq of cds	Length (nt)
*AmFoxP*	*GAGAAACCGCTGGACGTTTC*	NM_001104949	847
	*CGTTGCGCCGGAAGTAGCAG*		
*AmVGlut*	*GGCCCCCATTGCGTCACA*	XM_016914051.1	631
	*AATGCCAGCCACAACCAGAAACAGTA*		
*AmGad*	*AATGGTGAACGTCTGCTTCTGGTAT*	XM_391979	806
	*ACTTACGTGCTATGAGTATCCTTTG*		
*AmVAchT*	*CTCGGGCGCGTTGATAGACAGGAT*	XM_006562557.2	605
	*CGCCGAACACGTGGGGGAAGAA*		
*AmVMAT*	*AAGGCGTTGGTTCGTCGTGCTC*	XM_392061.6	855
	*CTTCTTTCGTTGGCGGTGCTCGTAA*		
*D2*-*like/AmDop3*	*CAGCGCATTCGTTAATCTGA*	NM_001014983.1	534
	*GCCCAGACCAACAGTATCGT*		

Primer sequences, reference sequence IDs and probe length are given. The AmFoxP-probe sequence containing plasmid was kindly given by Prof. Taketoshi Kiya, Kanazawa University (Japan). The AmGad primer pair was adopted from Kiya et al. [58]

PCR-amplified fragments were purified with a purification kit (Macherey–Nagel, Düren, Germany) and ligated into pGemT-easy plasmids (Promega, Wisconsin, USA) which were transformed in *E. coli* bacteria (Top 10). DNA-templates for RNA-probe synthesis were PCR-amplified with pGemt-easy M13 primers, detected by gel electrophoresis and purified again. SP6 and T7 polymerases (Roche, Mannheim, Germany) were used for in vitro transcription with Dig- and FITC-labeled UTP containing RNA Labeling Mix (Roche, Mannheim, Germany) and 200 ng of the cDNA template. Probes were purified with mini Quick Spin Columns (Roche, Mannheim, Germany), diluted 1:1 in formamid and stored at − 80 °C.

### Tissue preparation

Honeybee brains were quickly dissected in DEPC-treated water, immediately embedded in TissueTek^®^ (Sakura, Staufen, Germany), frozen on dry ice and kept at − 80 °C until use.

### Staining protocol—fluorescent double label

The double-label *ISH* (*dISH*) protocol was kindly provided by Prof. T. Kiya (Kanazawa University), published in Kiya and Kubo [58] and was slightly modified. Briefly, 12 µm frontal cryosections were fixed 15 min in 4% PFA in DEPC-treated water at room temperature (RT), washed in 0.1 M phosphate buffer pH 7.4 (PB: 18 mM NaH_2_PO_4_, 82 mM Na_2_HPO_4_), permeabilized and covered with the respective Dig- and FITC-labeled RNA probes. Per slide 0.3 µl *AmFoxP* and 1 µl neurotransmission marker probes were diluted in 150 µl hybridization buffer [58]. Slides were covered with coverslips (24 × 60 mm, Carl Roth, Karlsruhe, Germany) and hybridized overnight at 60 °C in a mineral oil bath. The next day, slides were washed sequentially in 5×, 2× and 0.2× saline-sodium citrate buffer pH 7.0 (20× SSC: 3 M NaCl, 0.3 M trisodium citrate), blocked in blocking reagent (Roche, Mannheim, Germany) and incubated 2 h at RT with anti-Dig-POD antibody (1:500, Roche, Mannheim, Germany). RNAse A treatment was not applied. After several washes, slides were incubated 15 min at RT in Cy5-labled tyramids (‘TSA-system’ by Perkin Elmer, Rodgau, Germany) according to the manufacturer’s protocol. Peroxidase was reduced by 3% hydrogen peroxide, the slides blocked again and incubated overnight at 4 °C with anti-FITC-POD (1:500, Roche, Mannheim, Germany). The next day, slides were washed and incubated in Cy3-labled tyramids (‘TSA-system’ by Perkin Elmer, Rodgau, Germany), counterstained with DAPI for 10 min at RT (1:20,000) and embedded in ‘Immu-Mount^™^’ (Thermo Fisher, Braunschweig, Germany).

Adjacent cryosections were incubated each with the *AmFoxP*-specific Dig-labeled probe and a second FITC-labeled probe specific for one of the four neurotransmitter markers, which were the honeybee (*Am*) *vesicular transporters* of either (1) *acetylcholine* (*AmVAchT*), (2) *glutamate* (*AmVGluT*) and (3) *monoamines* (*AmVMAT*) or (4) *glutamate decarboxylase* (*AmGad*) (Tables 2, 4).

We also performed one *dISH* with the FITC-labeled *AmVAchT* and a Dig-labeled *AmVGluT* probe on a newly emerged bee. Sample sizes for bees analyzed for neurotransmitter markers are listed in Table 1. For the analysis of the monoaminergic receptor *AmDop3* in the KC, one unmanipulated adult forager of undetermined age was analyzed using *dISH*.

Probes were chosen for *dISH* if two conditions were met: for each probe, the corresponding control (‘sense’) probe produced no specific staining and the ‘antisense’ probe labeled neurons known to express the particular neurotransmitter, e.g.: the octopaminergic VUMmx neurons located ventrally to the gnathal ganglia (GNG) for *AmVMAT* [53], the glutamatergic cortex of the optic lobes for *AmVGluT* [52], the inhibitory local interneurons and inhibitory projection neurons lateral and dorsal to the antennal lobes (AL) [50, 57, 94] as well as the neurons of the protocerebral-calycal tract (p.c.t.) [95, 96] for *AmGad* and the uniglomerular projection neurons (uPNs) of the antennal lobes [57, 97, 98] for *AmVAchT*.

### Chromogen single label

Tissue was dissected as described for *dISH* above but incubated with biotinylated (instead of fluorescently labeled) tyramids and DAB was used as the chromogen according to the manufacturer’s protocol (DAKO/agilent, Santa Clara, USA). All sense and antisense probes were first used in a single-label protocol to check for specificity.

### Immuno-labeling of AmFoxP protein

AmFoxP protein was detected using the custom-made AmFoxP^42kDa^ antiserum (RRID: AB_2722599). Antiserum specificity was shown previously [25, 32], immuno-labeling protocol, microscopy and data analysis was performed as described previously [25, 32]. Figure 7b was prepared from an immuno-labeled brain that was previously injected (in vivo) with Lucifer yellow (Life Technologies L453, CH lithium salt, MW 457.24; 5%) as described in [25].

### RT-qPCR and statistics

Animals were captured from an observation hive composed of marked bee as described above (‘unmanipulated’ group, see “Sampling method” section). They were immobilized on ice and decapitated. The head capsule was fixed on wax, the brain was exposed and covered with 0.1 M PBS (137 mM NaCl, 2.7 mM KCl, 8.1 mM Na_2_HPO_4_ (2H_2_O), 1.47 mM KH_2_PO_4_), the calyces were visually identified, removed with forceps and homogenized with a Teflon pestle and a glass homogenizer filled with 200 µl Trizol (Braunschweig, Germany). One sample consisted of 8–10 animals. The precision of the dissection was evaluated by staining brains with SYTOX Green diluted 1:2000 to reveal cell bodies (Fig. 14k, l). Total RNA was extracted and cDNA was synthetized from 1 µg of total RNA (see “Preparation of in situ hybridization probes” section). For the RT-qPCR experiments, 5 µl of diluted cDNA, 1 µl of the forward and reverse primers (10 µM, TIB Molbiol, Berlin, Germany), 10 µl Kapa Sybr Fast qPCR mastermix (PeqLab, Erlangen, Germany), 0.4 µl low Rox (50 nM) adjusted to 25 µl with water and analyzed on a Stratagene MX3000P (Agilent Technologies, Santa Clara, USA). Primers are listed in Table 3, *AmViaat* is the vesicular transporter for inhibitory amino-acids that is specific in vertebrates for GABA and glycine. This candidate was selected instead of *AmGad* because its expression levels can be better compared to the other vesicular transporters. *AmRpL32* and *AmGapDH* were chosen as reference genes. Primer efficiency was calculated on serial dilutions (10, 10^2^, 10^3^ and 10^4^) of the cDNA, primer interactions or the formation of unspecific products evaluated by melting curve analysis at the end of the amplification. On each plate, ‘no template controls’ (NTC) were analyzed by replacing cDNA with water for each parameter. Each sample was analyzed in triplicate. In Fig. 14l, the PCR profile was 2 min 95 °C, 40 amplification cycles 30 s at 95 °C, 30 s at 59 °C, 30 s at 72 °C and the melting curve analysis: 1 min 95 °C, 30 s at 55 °C, increasing to 95 °C and 30 s at 95 °C. The Cts were calculated by amplification based threshold. Fold change was calculated with the corrected amplification rate: e^−Ct^ (e = 10^−1/slope^) and the data were normalized to the geometric mean of the housekeeper genes, which did not vary over age groups (Kruskal–Walli statistic H(2) = 0.6, *p* = 0.74). Plots and statistics were performed with GraphPad Prism version 5.00 for Windows (GraphPad Software, La Jolla California USA) (RRID: SCR_015807). Data was tested for normal distribution using D’Agostino & Pearson omnibus normality test. Two means were compared for statistical differences using two-tailed Student’s t test.

**Table 3:** RT-qPCR primer list and amplification efficiency

mRNA	Forward 5′ 3′	Reverse 5′ 3′	Efficiency (%)
*AmVAchT*	AGGGCGTCGGTTCCGCTTTC	CAGCATCACTCCGTCCGCCA	89.7
*AmVGluT*	AACGCCCCGTGAGGGTAGCA	GACGCAATGGGGGCCGTTCA	96.3
*AmVMAT*	ATTGTCGGCCCCCTCACCCA	GAGCACGACGAACCAACGCC	99.7
*AmVIAAT*	CGCCGTATTGCGAGGCGGTT	CGCGTTGTCCAGTCGTCGTGT	99.8
*AmRpL32*	TGTGCTGAAATTGCTCATGGGGG	AGAACGTAACCTTGCACTGGCATAA	100.3
*AmGapDH*	CGGTTTTGGCCGTATTGGCCGT	AATGGCAACAACCTGAGCACCGAA	99.4

## Results

### AmFoxP expressing neuron populations (clusters and groups)

We determined which of the four investigated neurotransmitter markers, *AmVAchT*, *AmVGLuT*, *AmVMAT* and *AmGad*, were co-expressed in the 11 AmFoxP neuron populations. We identified these population previously based on mRNA in situ hybridization and immunoreactivity to a custom-made antiserum (‘anti-AmFoxP^42kDa^’) [25, 32]. The anatomical nomenclature follows Ito et al. [99], Table 5. For ease of reading, we will use the terms AmFoxP neuron, AmFoxP cluster and AmFoxP group. We previously showed these cells to produce both *AmFoxP* mRNA and AmFoxP protein [25, 32]. Here we use immunohistochemistry and in situ hybridization (ISH) interchangeably. Neurons that were neither immunoreactive to the AmFoxP^42kDa^ antiserum nor labeled by the *AmFoxP* ISH probe are referred to as AmFoxP-negative.

**Fig. 1:**
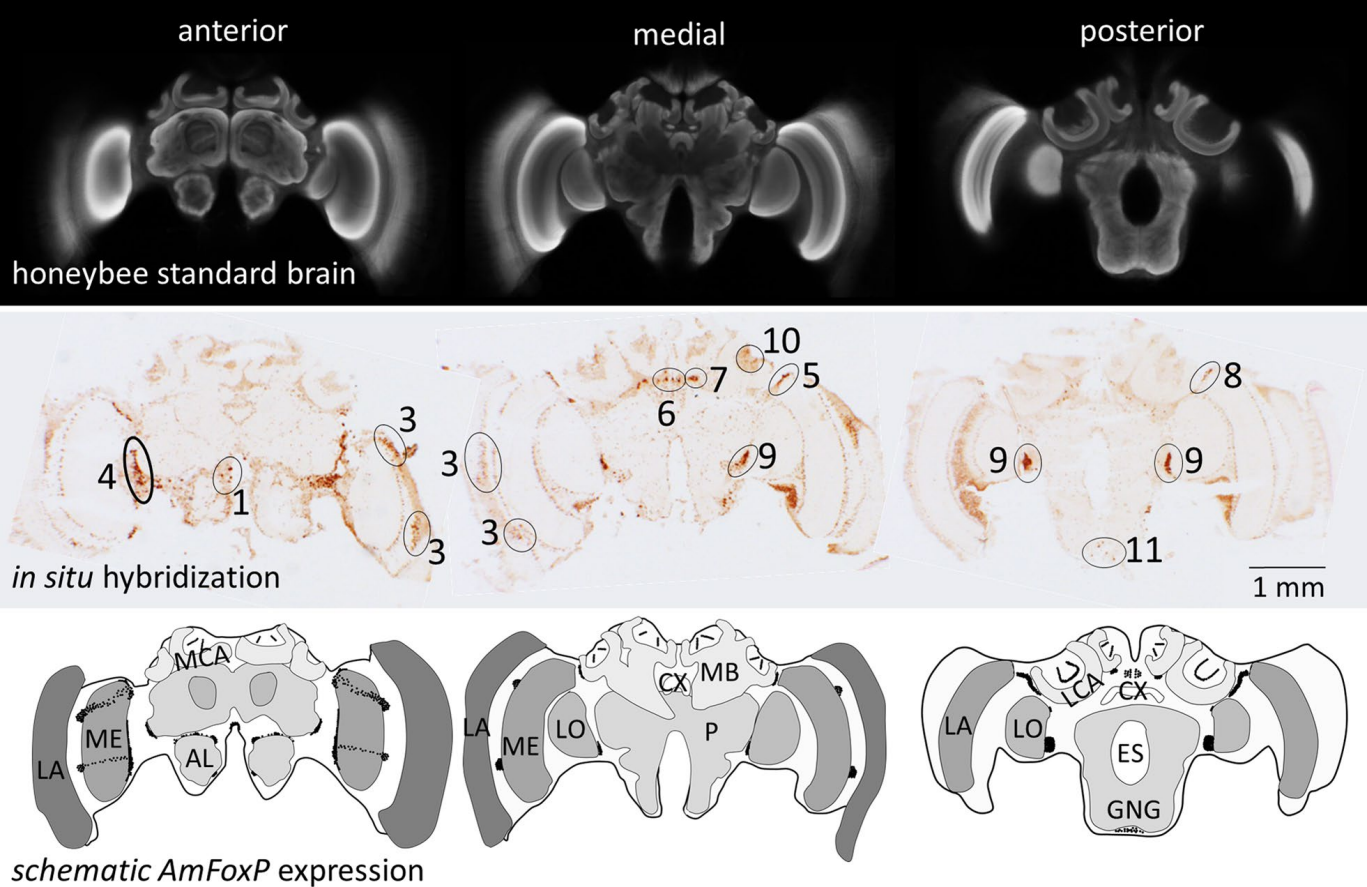
Locations of the 11 *AmFoxP* neuron populations in the honeybee brain. Top: single confocal sections from the Honeybee Standard Brain Atlas [100] depicting representative sections at the anterior, medial and posterior level. Middle: single-label in situ hybridization (ISH) with the *AmFoxP* antisense probe on three honeybee brain cryosections (12 µm) corresponding to the anterior–posterior levels depicted in the top row. Clusters are indicated by numbers (see Table 4). Bottom: schematic representation of the AmFoxP neuron populations (black dots) at corresponding levels. Anatomical abbreviations are listed in Table 5

We previously described seven AmFoxP ‘clusters’, which consist of densely packed neurons and four ‘groups’, in which the AmFoxP neurons are more broadly distributed ([25], Fig. 1, Table 3). In the present study, we found that each of the seven AmFoxP clusters homogeneously co-expressed only one of the used neurotransmitter markers. In contrast, all AmFoxP groups co-expressed two or even three of the transmitter markers, but not *AmVMAT*. This difference between clusters and groups was observed in all treatment groups and ages.

### Neurotransmitter expression

All four neurotransmitter markers used in the present study were detected in cortical areas around all major neuropils (Figs. 2, 3, 4, 5, 6, 7, 8, 9, 10, 11, 12, 13).

**Fig. 2:**
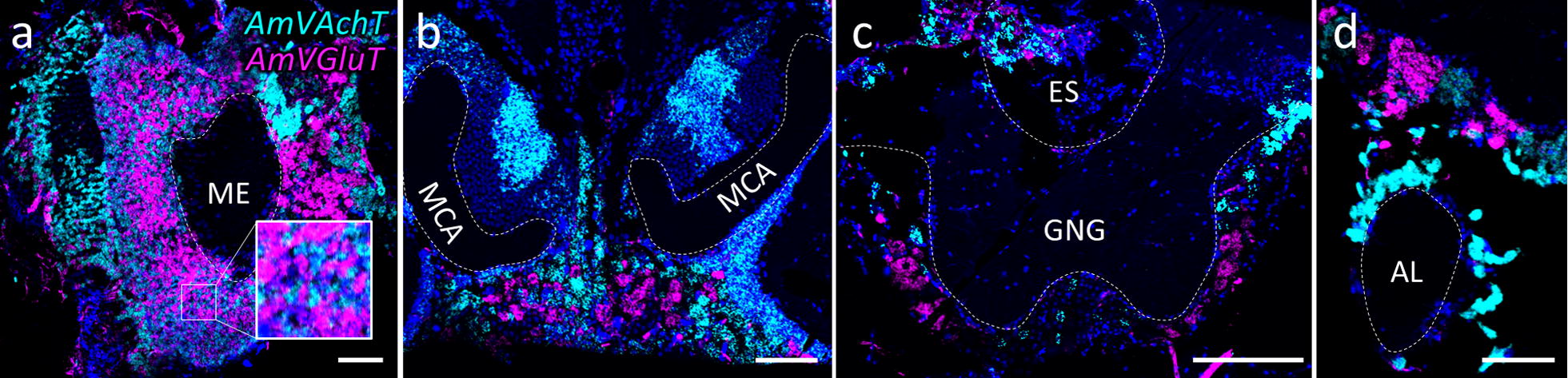
AmVAchT (cyan) and *AmVGluT* (magenta) do not co-localize within the same neurons. Double ISH (dISH) stainings on 12 µm cryosections from the brain of a newly emerged worker honeybee depict somatic areas surrounding different neuropils: **a** the area around the medulla (ME), **b** the area ventral to the medial calices (MCA) in the posterior brain, **c** the area ventral and lateral to the gnathal ganglia (GNG) and **d** the area around the antennal lobes (AL). Rectangular inset in **a** shows higher magnifications of small boxed region. Scale bars: 100 µm

**Fig. 3:**
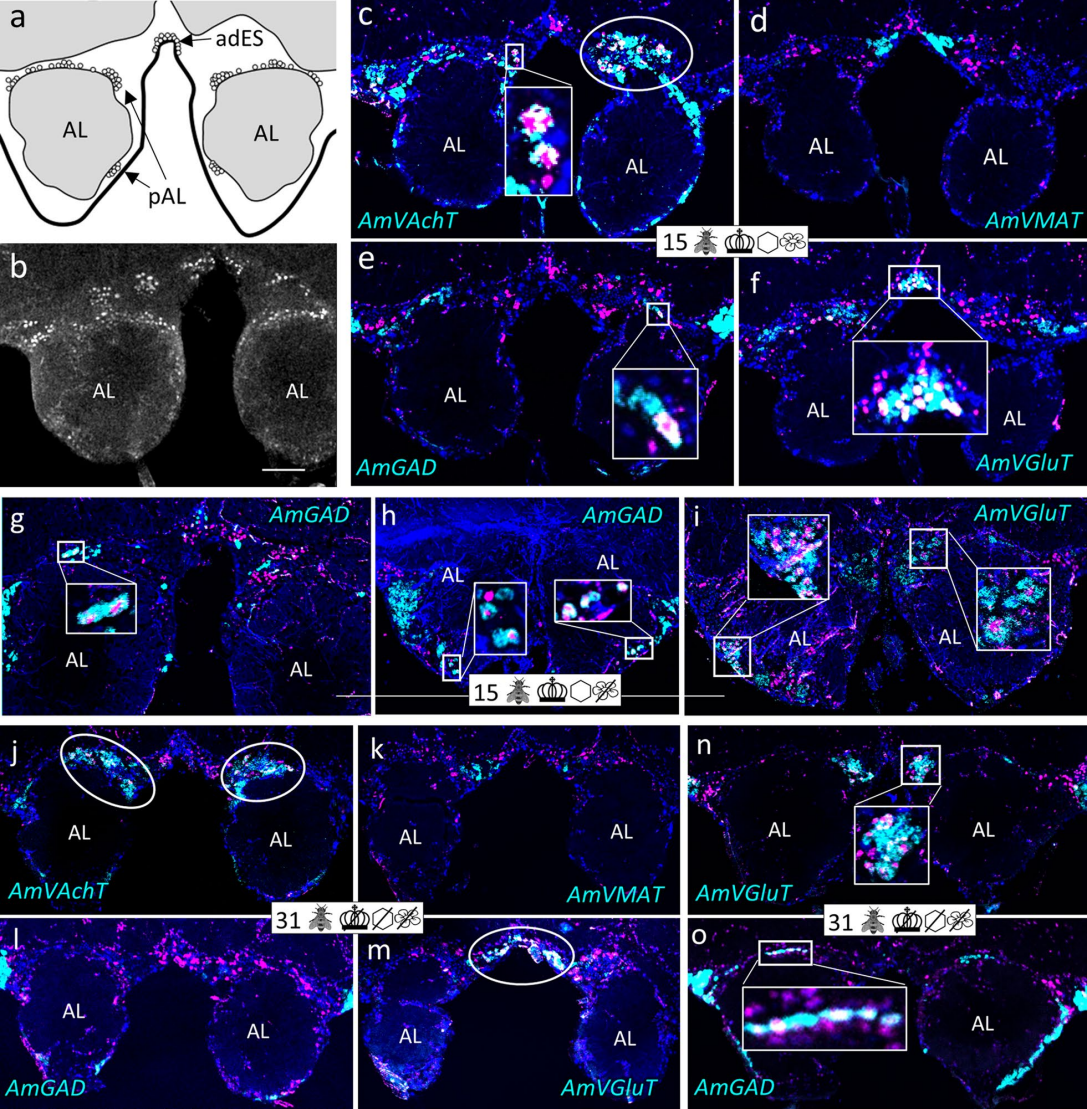
Group ‘around (peri) antennal lobe’ (pAL) and cluster ‘anteriodorsal to the esophageal hole’ (adES). **a** Schematic drawing of the honeybee AL. AmFoxP neurons are depicted as empty circles, arrows point to the two neuron populations. **b** Confocal image (2 µm confocal section) of a pupal brain stained with the AmFoxP^42kDa^ antiserum (white label). **c**–**o** dISH stainings show *AmFoxP* transcript (magenta), neurotransmitter marker transcript (cyan) and DAPI-stained nuclei (blue). Large rectangular insets show higher magnifications of small boxed regions, with white label reflecting co-expressing neurons. Some double-labeled neurons are also visible in circled areas in **c**, **j**, **m**. **c**–**f** Adjacent cryosections of a 15 days old individual from the ‘unmanipulated’ condition. **g**–**i** Adjacent cryosections of a 15 days old individual from the ‘honeycomb’ condition, anterior (**g**) and posterior (**h**, **i**). **j**–**o** Adjacent cryosections of a 31 days old individual from the ‘incubator’ condition, anterior (**j**–**m**) and posterior (**n**, **o**). Pictograms refer to the conditions outlined in Table 1. Anatomical abbreviations see Table 5. Scale bar **b**: 100 µm, as orientation for all other panels

**Fig. 4:**
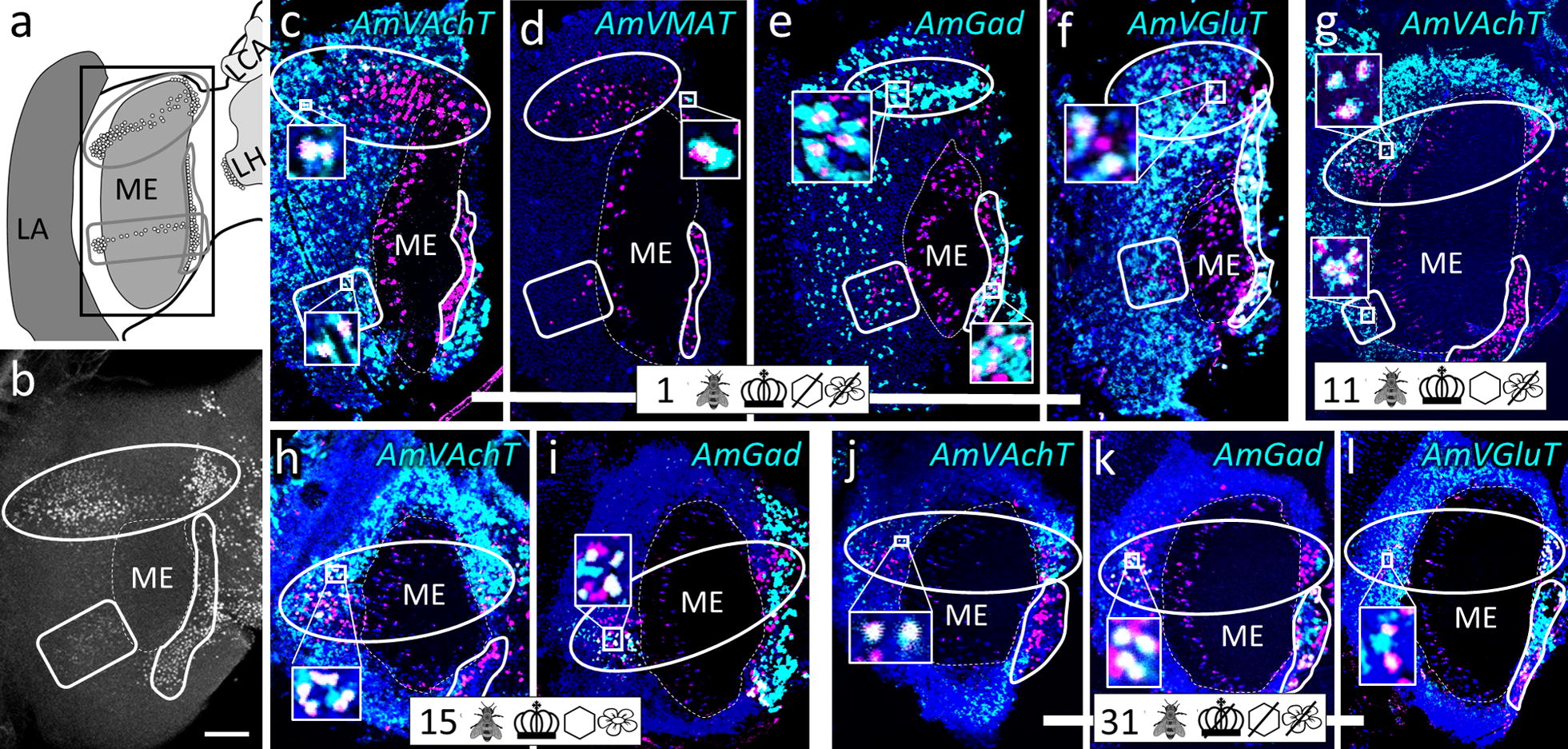
Group ‘around (peri) medulla’ (pME). **a** Honeybee brain schematic drawing focuses on the ME. AmFoxP neurons are depicted as empty circles which surround the ME in three subsets: a dorsal (gray circle) and a ventral (gray rectangle with rounded corners) subset wrap around the anterior ME, a third subset (outlined in gray) is bordering the ME medially in a thin strip. The black rectangle indicates the localization of panels (**c**–**l**). **b** Confocal image (60 µm confocal stack) of a pupal brain stained with the AmFoxP^42kDa^ antiserum (white label). The outlined areas refer to the AmFoxP subsets as in **a**, the ME is indicated with a dashed line. **c–l** dISH stainings showing *AmFoxP* transcript (magenta), neurotransmitter transcript (cyan) and DAPI-stained nuclei (blue). Rectangular insets show co-localization of the two probes, resulting in white label. **c**–**f** Adjacent cryosections of a newly emerged individual. **g** Cryosections of an 11 days old individual from the ‘honeycomb’ group. **h**, **i** Adjacent cryosections of a 15 days old individual from the ‘unmanipulated’ group. **j**–**l** Adjacent cryosections of a 31 days old individual of the ‘incubator’ group. For explanations of pictograms see Table 1, for anatomical abbreviations see Table 5. Scale bar **b**: 100 µm, as orientation for all other panels

**Fig. 5:**
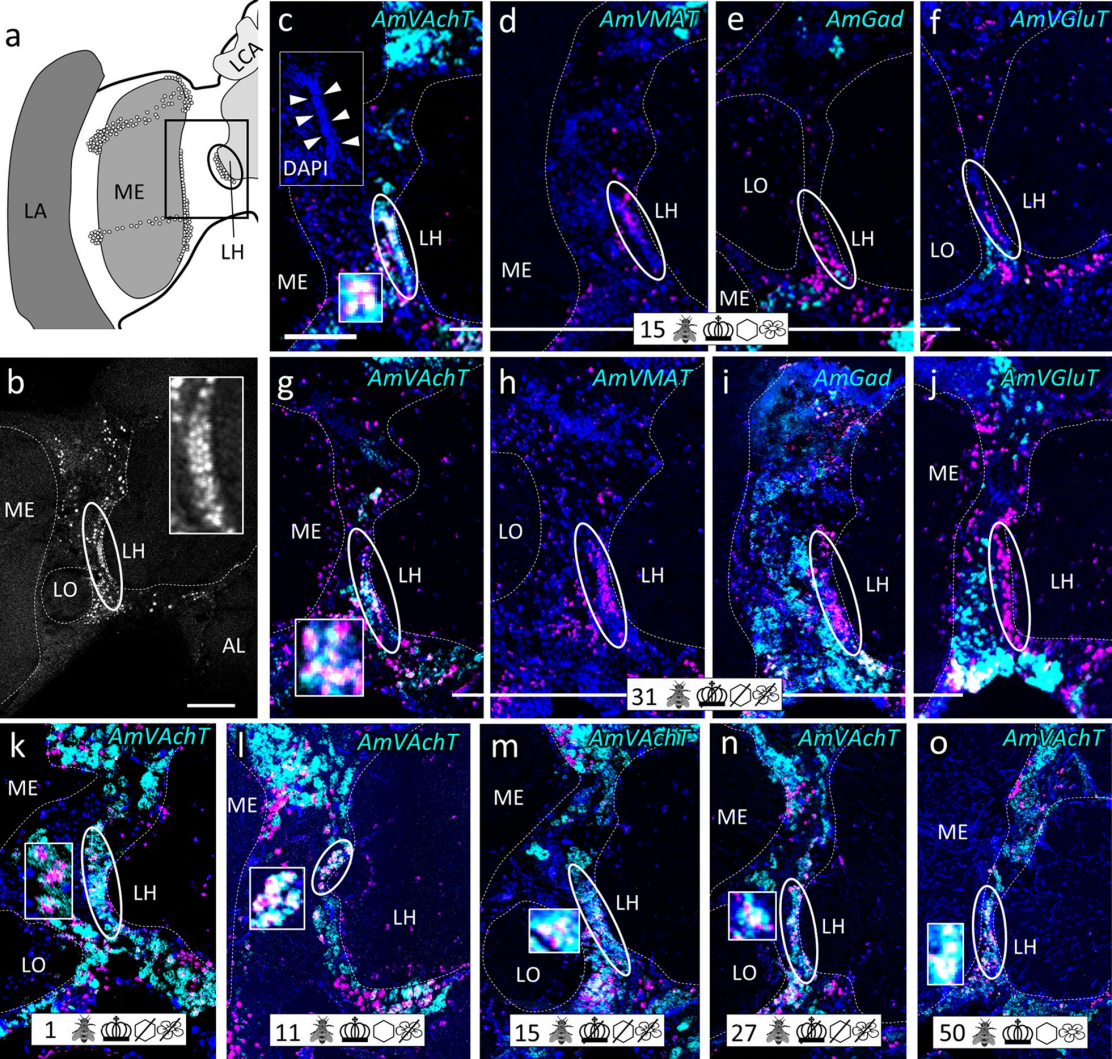
Cluster ‘lateral to the lateral horn’ (lLH). **a** Honeybee brain schematic drawing focuses on the LH and ME. AmFoxP neurons are indicated with small empty circles. Rectangle indicates the localization of panels (**c**–**o**). **b** Confocal image (2 µm confocal section) of a forager brain stained with the AmFoxP^42kDa^ antiserum (white label). **c**–**o** dISH stainings showing *AmFoxP* transcript (magenta), neurotransmitter transcript (cyan) and DAPI-stained nuclei (blue) on 12 µm brain cryosections. Small rectangular insets in **c**, **g**, **k**–**o** show co-localization of the two probes. Larger rectangular insets in **b**, **c** show densely-packed small nuclei of the lLH, stained with AmFoxP^42kDa^ antiserum (**b**) and DAPI (**c**). **c**–**f** Adjacent cryosections of a 15 days old individual from the ‘unmanipulated’ group. **g**–**j** Adjacent cryosections of a 31 days old individual from the ‘incubator’ group. **k** Newly emerged individual. **l** 11 days old individual from the ‘honeycomb’ group. **m** 15 days old individual from the ‘incubator’ group. **n** 27 days old individual of the ‘incubator’ group. **o** Cryosection of a 50 days old individual from the ‘unmanipulated’ group. For explanations of pictograms see Table 1, for anatomical abbreviations see Table 5. Scale bar **b**, **c**: 100 µm, as orientation for all other panels

**Fig. 6:**
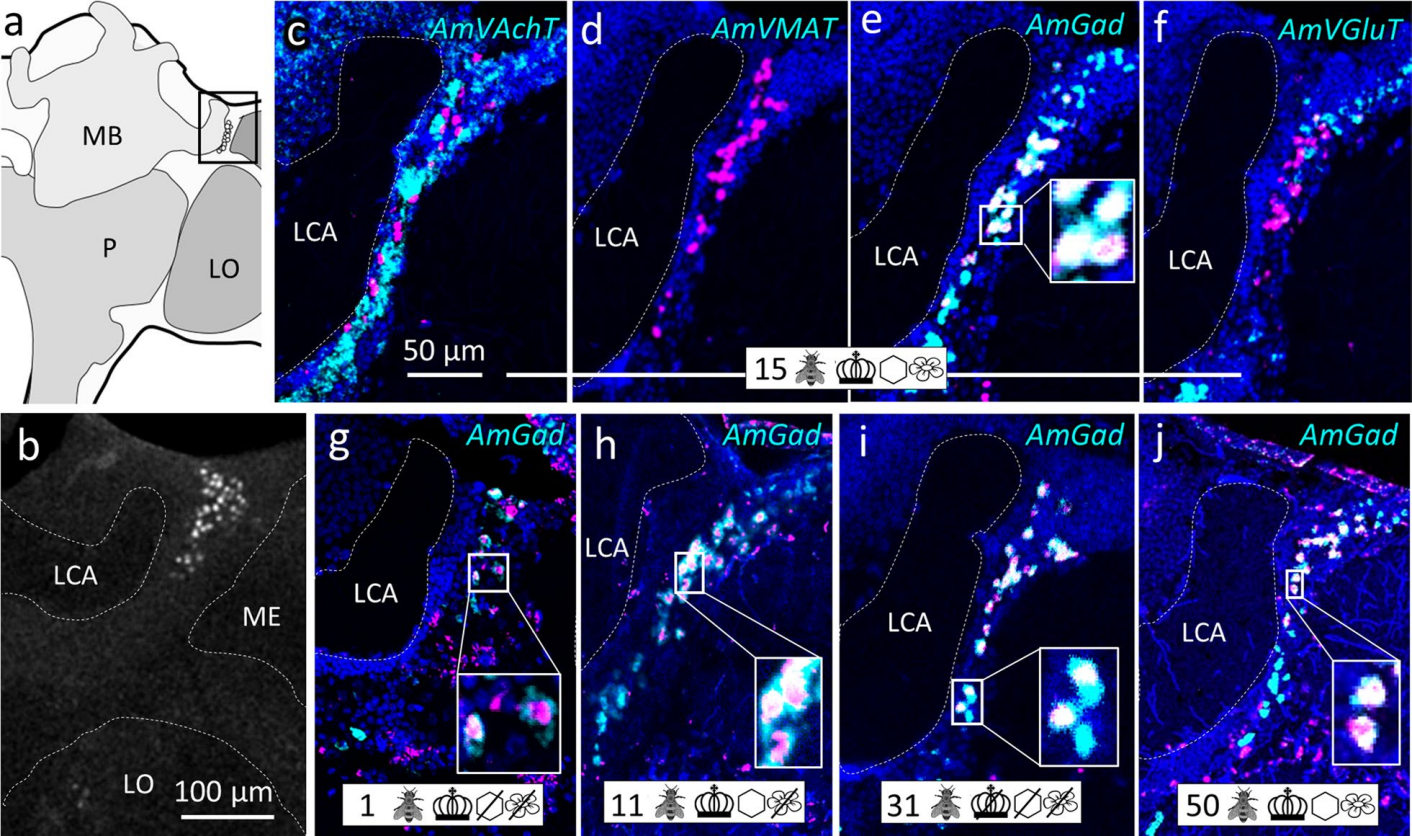
Cluster anteriolateral to the lateral calyx (alLCA). **a** Schematic drawing of the medial level of one hemisphere. AmFoxP neurons are indicated by small empty circles within the boxed area which indicates the localization of panels (**b**–**j**). **b** Confocal image (2 µm confocal section) of a pupal brain stained with the AmFoxP^42kDa^ antiserum (white label). **c**–**j** dISH stainings showing *AmFoxP* transcript (magenta), neurotransmitter transcript (cyan) and DAPI-stained nuclei (blue) on 12 µm cryosections. Rectangular insets show magnified boxed area with co-localization of the two probes. **c**–**f** Adjacent cryosections of a 15 days old individual from the ‘unmanipulated’ group. **g** Cryosections of a newly emerged individual. **h** Cryosection of an 11 days old individual from the ‘honeycomb’ group. **i** Cryosections of a 31 days old individual of the ‘incubator’ group. **j** Cryosection of a 50 days old individual of the ‘control’ group. For explanations of pictograms see Table 1, for anatomical abbreviations see Table 5. Scale bars as indicated. Scale bar in **c** as orientation for the panels (**d**–**j**)

**Fig. 7:**
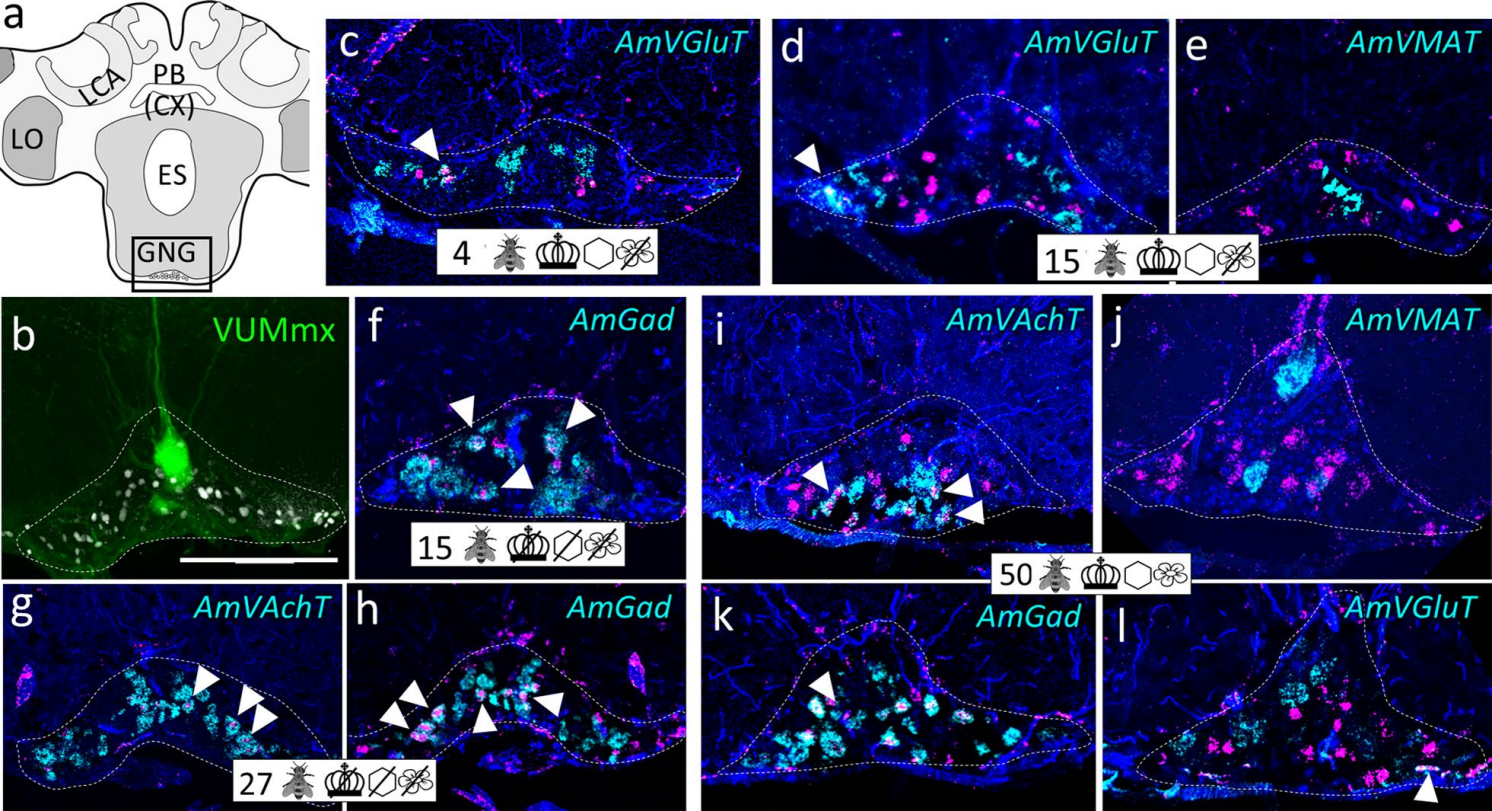
Group ventral to the GNG (vGNG). **a** Honeybee brain scheme focusing on the posterior brain. AmFoxP neurons are indicated with white circles within the boxed area which indicates the localization of panels (**b**–**l**). **b** Confocal image (60 µm confocal stack) of a forager brain backfilled with lucifer yellow (green) and subsequently stained with the AmFoxP^42kDa^ antiserum (white label). The large green-labeled neuron lying on the brain midline is a VUMmx neuron. **c**–**l** dISH stainings show *AmFoxP* transcript (magenta), neurotransmitter transcript (cyan) and DAPI-stained nuclei (blue) on 12 µm cryosections. White arrowheads point to neurons with co-localized probe signal. **c** Cryosections of a 4 days old individual from the ‘honeycomb’ group. **d**, **e** Adjacent cryosections of a 15 days old individual from the ‘honeycomb’ group. **f** Cryosections of a 15 days old individual from the ‘incubator’ group. **g**, **h** Adjacent cryosections of a 27 days old individual from the ‘incubator’ group. **i**–**l** Adjacent cryosections of a 50 days old individual from the ‘control’ group. For explanations of pictograms see Table 1, for anatomical abbreviations see Table 5. Scale bar **b**: 100 µm, as orientation for all other panels

**Fig. 8:**
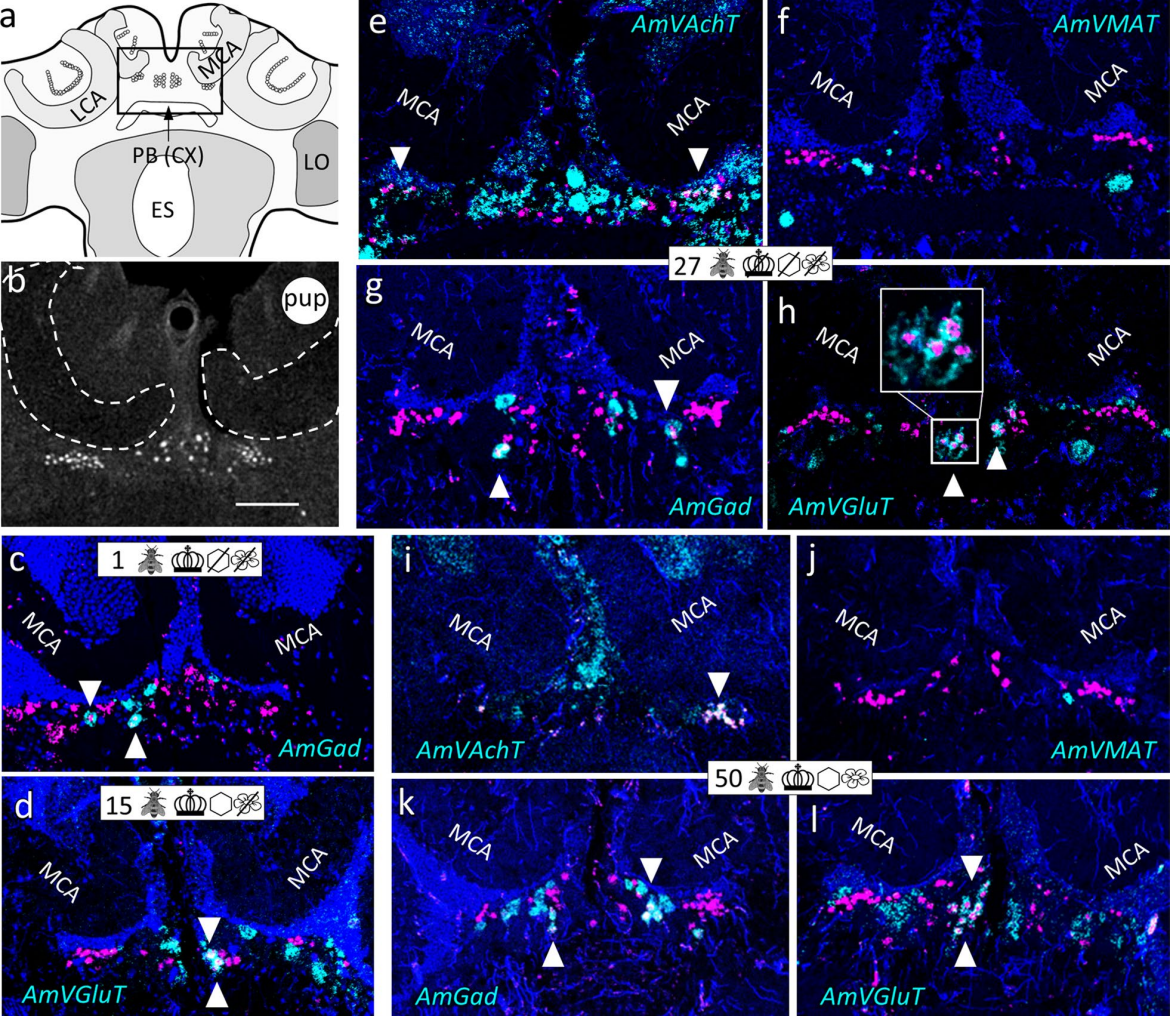
Cluster ventral to the medial calyx (vMCA) and group dorsal to the central complex (dCX). **a** Honeybee brain schematic drawing focuses on the posterior brain. AmFoxP neurons are depicted as small empty circles. Rectangle indicates localization of panels (**b**–**l**). **b** Confocal image (2 µm confocal section) of a pupal brain stained with the AmFoxP^42kDa^ antiserum (white label). **c**–**l** dISH stainings show *AmFoxP* transcript (magenta), neurotransmitter transcript (cyan) and DAPI-stained nuclei (blue) on 12 µm cryosections. Arrowheads point to neurons that co-express *AmFoxP* and a neurotransmitter. **c** Cryosection of a newly emerged individual. **d** Cryosection of a 15 days old individual from the ‘honeycomb’ group. **e**–**h** Adjacent cryosection of a 27 days old individual from the ‘incubator’ group. **i**–**l** Cryosection of a 50 days old individual from the ‘control’ group. For explanations of pictograms see Table 1, for anatomical abbreviations see Table 5. Scale bar **b**: 100 µm, as orientation for all other panels

**Fig. 9:**
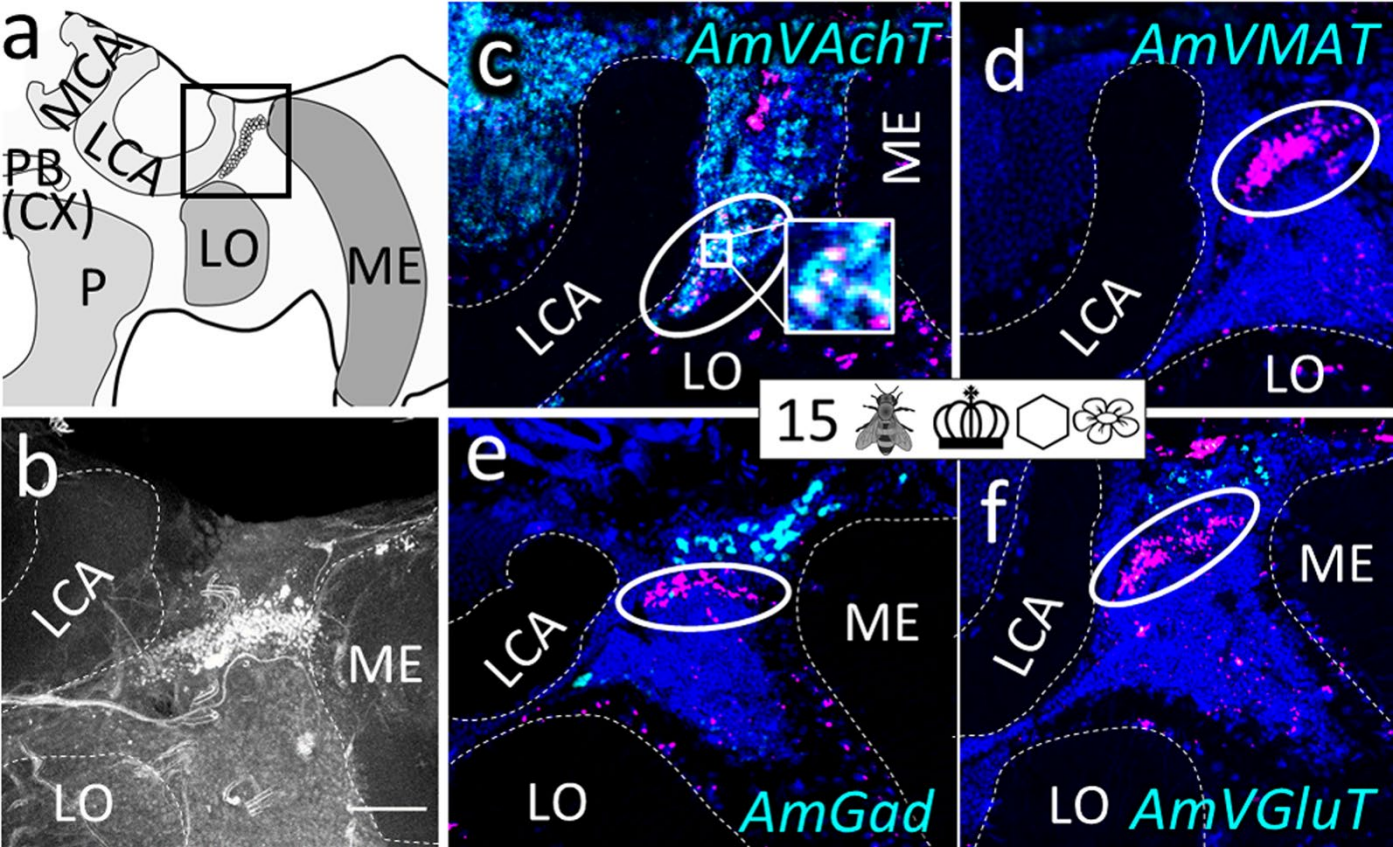
Cluster posteriolateral to the lateral calyx (plLCA). **a** Honeybee brain schematic drawing of the right hemisphere in the posterior brain. AmFoxP neurons are indicated with small white circles within the boxed area which indicates the localization of panels (**b**–**f**). **b** Confocal image (60 µm confocal stack) of a forager brain stained with the AmFoxP^42kDa^ antiserum (white label). **c**–**f** dISH stainings show *AmFoxP* transcript (magenta), neurotransmitter transcript (cyan) and DAPI-stained nuclei (blue) on 12 µm adjacent cryosections of a 15 days old individual from the ‘unmanipulated’ group. Inset in **c** shows magnification of small boxed area with co-localization of the two probes. For explanations of pictograms see Table 1, for anatomical abbreviations see Table 5. Scale bar **b**: 100 µm, as orientation for all other panels

**Fig. 10:**
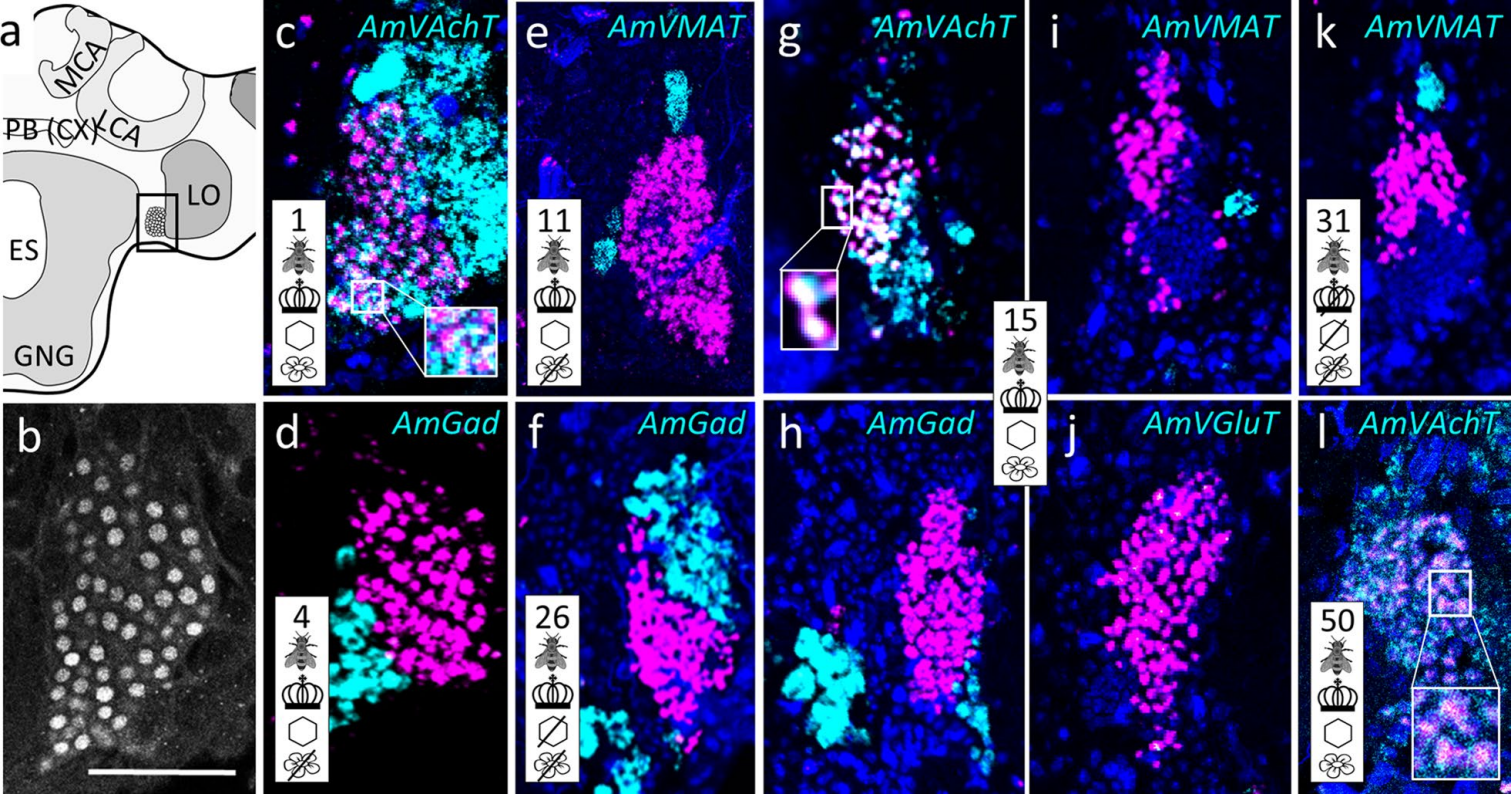
Cluster medioventral to the lobula-posterior part (mvLO-p). **a** Honeybee brain schematic drawing focuses on a posterior brain hemisphere. AmFoxP neurons are indicated by small empty circles within the boxed area which indicates localization of panels (**b**–**j**). **b** Confocal image (2 µm confocal section) of a forager brain stained with the AmFoxP^42kDa^ antiserum (white label) shows the mvLO-p. **c**–**l** dISH stainings showing AmFoxP transcript (magenta), neurotransmitter transcript (cyan) and DAPI-stained nuclei (blue) on 12 µm cryosections. **c** Cryosection of a newly emerged individual. **d** Cryosection of a 4 days old individual from the ‘honeycomb’ group. **e** Cryosection of a 11 days old individual of the ‘honeycomb’ group. **f** Cryosection of a 26 days old individual of the ‘mini-cage’ group. **g**–**j** Adjacent cryosections of a 15 days old individual from the ‘unmanipulated’ group. **k** Cryosection of a 31 days old individual of the ‘incubator’ group. **l** Cryosection of a 50 days old individual of the ‘unmanipulated’ group. Inset in **c**, **g**, **l** show magnification of boxed area with co-localization of the two probes. For explanations of pictograms see Table 1, for anatomical abbreviations see Table 5. Scale bar **b**: 50 µm, as orientation for all other panels

**Fig. 11:**
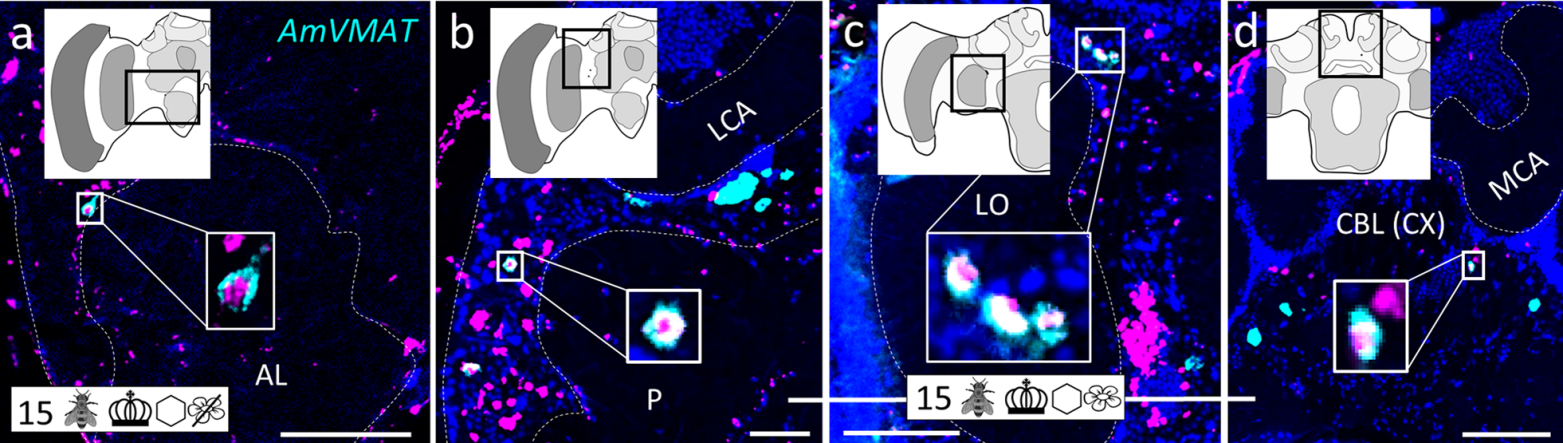
Individudal ‘MF’ neurons (monoaminergic AmFoxP neurons) that co-express AmFoxP and *AmVMAT*. **a**–**d** dISH stainings showing AmFoxP transcript (magenta), *AmVMAT* (cyan) and nuclei (blue) in honeybee brain cryosections, anterior (**a**) to posterior (**d**). Insets outlined by white boxes show co-localization of the two probes. Locations in the brain where photomicrographs were taken are outlined (black line) in the schematic drawing in the insets at the top of each panel. **a** ‘MF1’ neurons dorsolateral to the antennal lobe (AL) of a 15 days old individual from the ‘encaged honeycomb’ group. **b** ‘MF2’ neuron medial to the medulla (ME). **c** ‘MF3’ neurons dorso-medial to the lobula (LO). **d** ‘MF4’ neurons close to the fan-shaped body (FB) of the central complex (CX). **b**–**d** Cryosections of a 15 days old individual from the ‘control’ group. For explanations of pictograms see Table 1, for anatomical abbreviations see Table 5. Scale bars: 100 µm

**Fig. 12:**
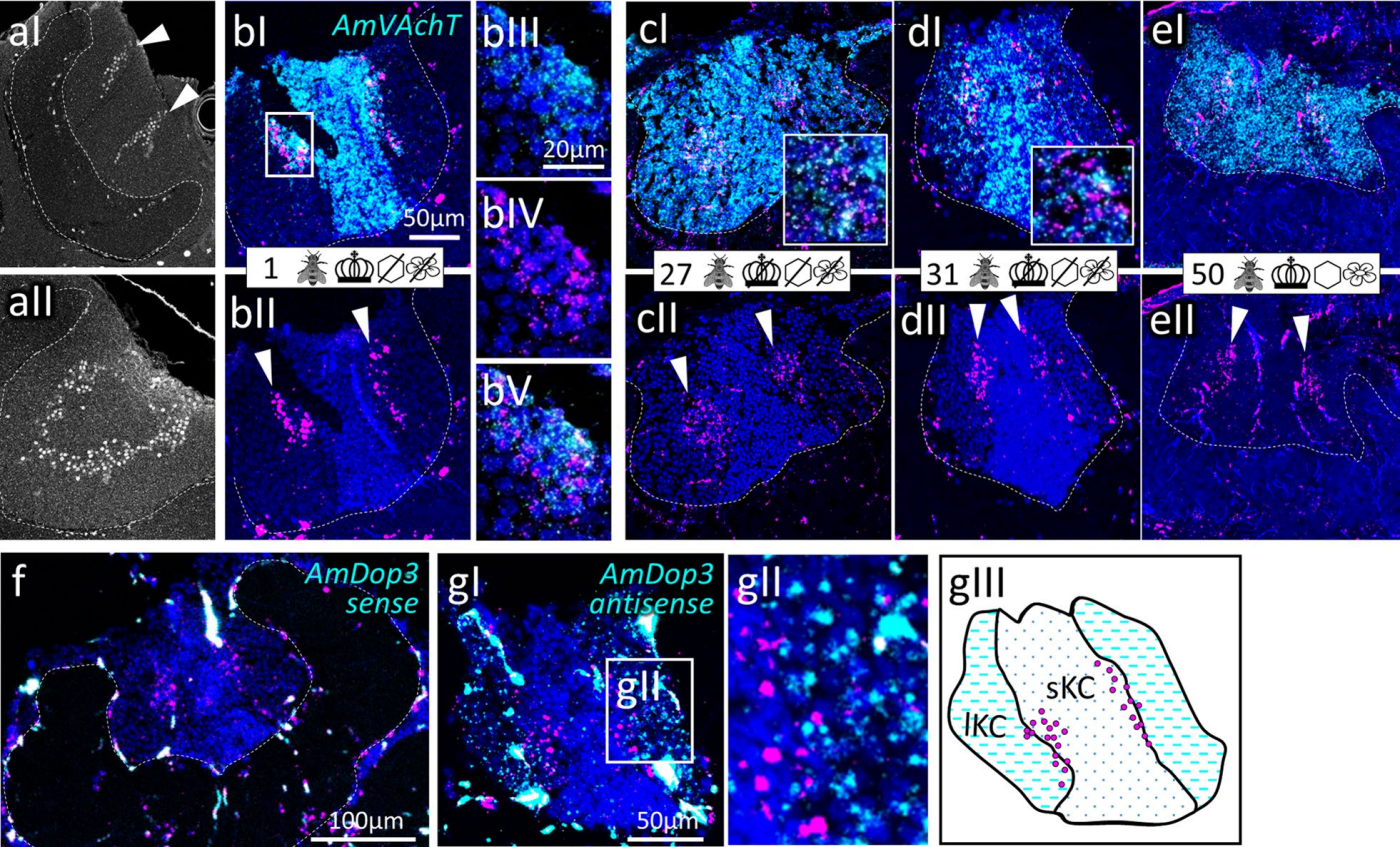
AmFoxP expressing Kenyon cells (KC) of the mushroom bodies. **a** Single confocal sections (1 µm) show the KC of a whole-mount pupal brain stained with the AmFoxP^42kDa^ antiserum (white label), (aI) anterior, (aII) posterior. The calyx is encircled with a dashed line. White arrowheads point to the elongated AmFoxP neuron clusters (mKC). **b**–**e** ISH stainings show *AmFoxP* transcript (magenta) and *AmVAchT* transcript (cyan) on 12 µm cryosections of individuals taken from the different treatment groups. Age and treatment group are indicated with symbols that can be seen in Table 1. Magnifications of inset in **b** is shown in panels (**bIII–V**). The two transcripts of *AmFoxP* and *AmVAchT* are mostly co-expressed in the same cells. **f**, **g** dISH stainings show expression of the D2-like dopamine-receptor *Amdop3* (cyan) and *AmFoxP* (magenta). The *Amdop3* sense probe (control) did not show any (unspecific) staining (**f**). *Amdop3* is mostly expressed in the lKC but there was a small overlap with the *AmFoxP*-expressing mKC (**gI**–**gIII**). Inset in **gI** is shown magnified in **gII**. **gIII** shows a schematic of **gI**. Blue: nuclear staining (DAPI). Sizes of scale bars are indicated

**Fig. 13:**
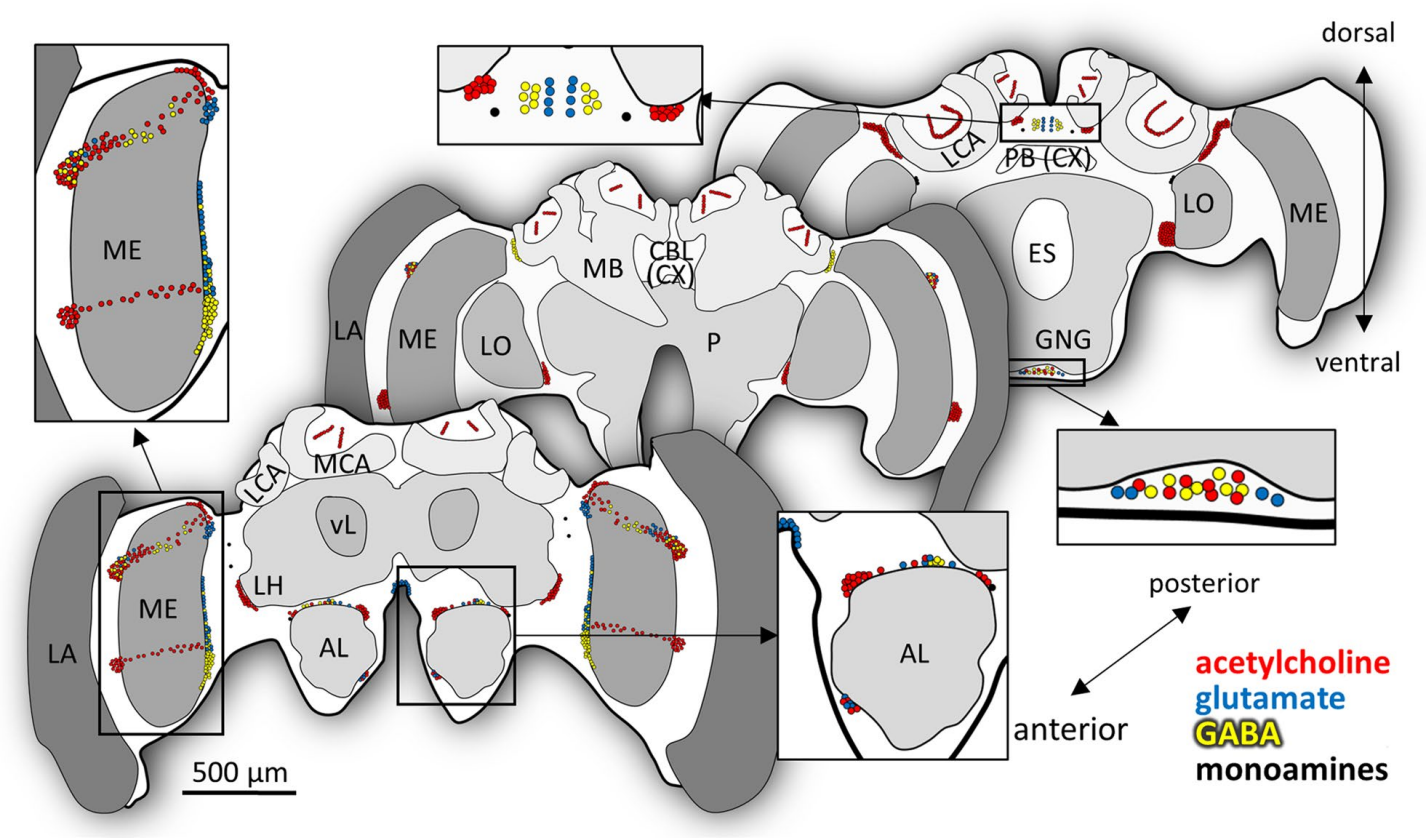
Schematic summary of the neurotransmitter profiles of the AmFoxP neurons. Frontal views of anterior to posterior levels of the honeybee brain. Front to back: Small colored circles represent AmFoxP neurons, different colors indicate different neurotransmitters: glutamate (blue), acetylcholine (red), GABA (yellow), monoamines (black). For anatomical abbreviations see Table 5. Scale bar as indicated

The mRNA of *AmVAchT* and *AmVGluT* as well as *AmGad* were very abundant in cortical areas surrounding the antennal (AL) (Fig. 3) and optic lobes (OL) (Fig. 4), as well as the gnathal ganglia (GNG) (Fig. 7). The markers of the two excitatory neurotransmitters *AmVAchT* and *AmVGluT* had similar expression patterns. However double labeling showed that both *AmVAchT* and *AmVGluT* mRNAs were not co-expressed in the same neurons (Fig. 2).

*AmVMAT* expression was scarce and found in cell clusters (Figs. 3d, 5d) as well as in single cells (Figs. 4d, 7d, l, 8d, h, 10e, i, k, 11).

#### Cholinergic AmFoxP neurons

**Fig. 14:**
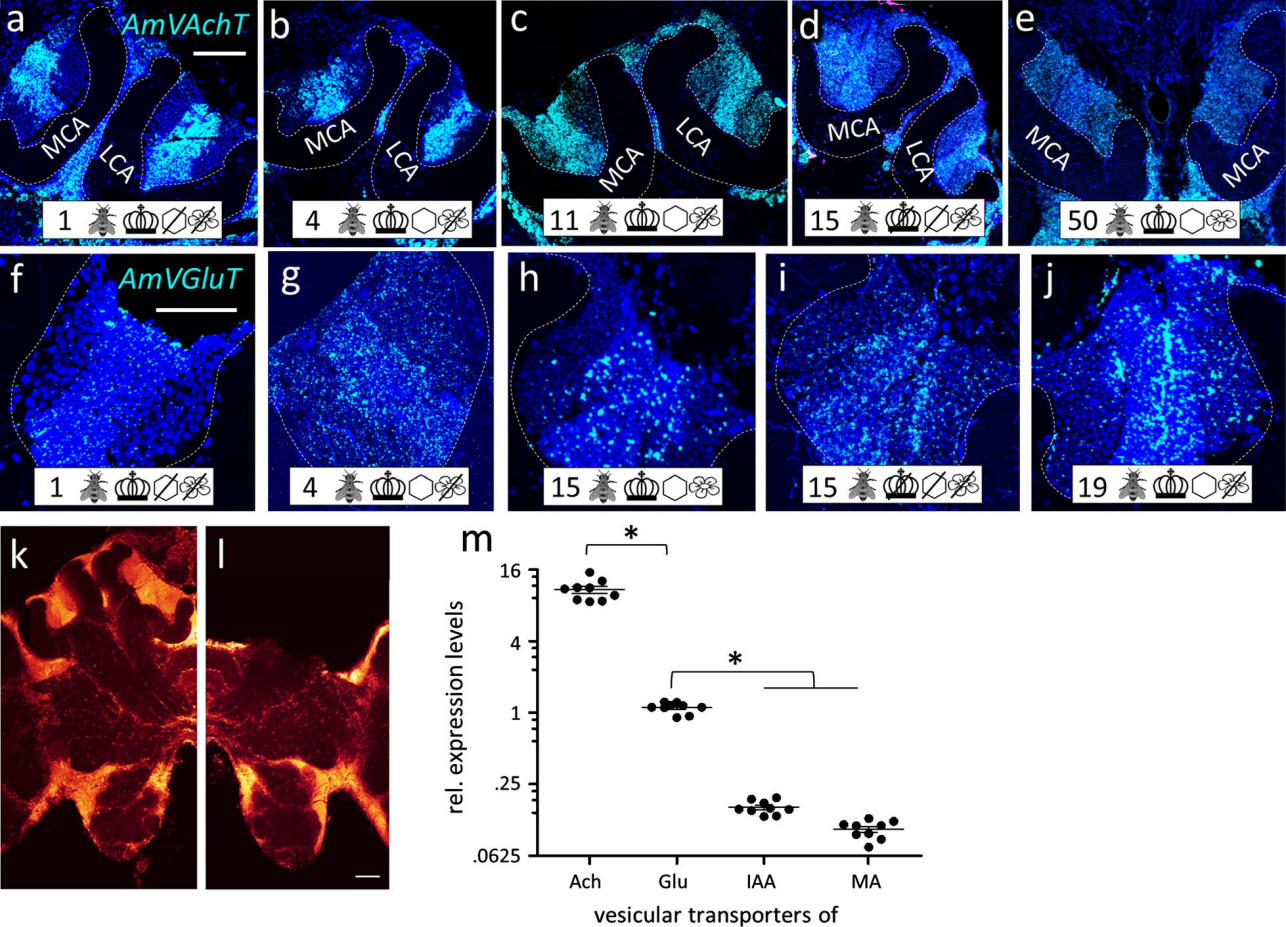
*AmFoxP* and *AmVGluT* mRNA expression in the KC. **a**–**j** ISH stainings on 12 µm cryosections of individuals taken from the different treatment groups (symbols, Table 1) show *AmVAchT* (**a**–**e**) and *AmVGluT* (**f**–**j**) expression in different KC subpopulations. *AmVAchT* was restricted primarily, in workers aged younger than 5 days, to the sKC and mKC (**a**, **b**). With increasing age, the lKC also started expressing *AmVAchT* (**c**–**e**). Simultaneously, the expression intensity in the sKC decreased until, in a very old forager (50 days) there was no more difference between the sKC/mKC and the lKC detectable (**e**). **f**–**j** Very low *AmVGluT* signals in the sKC/mKC were detected in almost half of the samples (all shown here). Age and treatment groups are indicated with symbols that can be seen Table 1. **k**, **l** Two different forager brains (100 µm sections) before (**k**) and after (**l**) calyx dissection for RT-qPCR experiments (nuclear staining with SYTOX green). Scale bars: 100 µm, in **a**, **f** as orientation for the panels (**b**–**e**) and (**g**–**j**). **m** Relative expression levels of vesicular transporters in calyx extracts, measured by RT-qPCR. In adult foragers *AmVAchT* levels were ten times higher than *AmVGluT* levels (paired Student’s t(8) = 13.1, *p* < .0001). *AmIAAT* and *AmVMAT* levels were 7–11 times lower than *AmVGluT* levels (*AmVIAAT*: paired Student’s t(8) = 30, *p* < .0001, *AmVMAT*: paired Student’s t(8) = 27.4, *p* < .0001)

*AmVAchT* was detected in large cell clusters dorsal and dorso-lateral to the AL (Fig. 3c)—most likely uniglomerular projection neurons (uPNs) [57], in cortical areas around the OL and in the KC (Fig. 14a–e). The cholinergic marker was co-expressed in five of the seven AmFoxP clusters (Table 4), i.e. the lLH (Fig. 5), the plLCA (Fig. 6), the vMCA (Fig. 8), the large mvLO (Fig. 10) and the mKC (Fig. 12b–e). The mvLO cluster has a higher cell density than the surrounding tissue, clearly visible in nuclear DAPI staining (Fig. 10i, k). However, by AmFoxP labeling, this cluster could be further subdivided in two subdivisions, an anterior (mvLO-a) and a posterior (mvLO-p) part; each subdivision projects to a different brain area, as described previously [25]. Both subdivisions expressed *AmVAchT.* The mvLO-a and mvLO-p are separated by a layer of AmFoxP-negative neurons that expressed *AmGad* (Fig. 10f). *AmVAchT* was also clearly expressed in the region of the mKC and coincided with *AmFoxP* expression. However, due to the subcellular punctate staining in the KC (Fig. 12bIII–V), the two colors representing *AmFoxP* and *AmVAchT* did not coincide to the same extent as in other neurons. We therefore considered KC to co-express two probes as long as any punctate label was located within the area of a DAPI-positive nucleus, which was the case for *AmVAchT* in essentially all *AmFoxP*-expressing KC (but different in the *AmDop3*/*AmFoxP dISH*, Fig. 12g). In the AmFoxP groups, with less densely clustered cells and co-expression of more than one neurotransmitter, *AmVAchT* was expressed in neurons around the AL (pAL, Fig. 3c, j), around the medulla (pMe, Fig. 4c, g, j) and ventral to the GNG (vGNG, Fig. 7c, g) but not dorsal to the central complex (dCX, Fig. 8). The pME could be further subdivided in at least three local groups. Two of them wrap around the anterior medulla; one ventrally and one dorsally (Fig. 4a–g). The ventral AmFoxP neurons co-expressed only *AmVAchT* (Fig. 4c, g), whereas the dorsal one co-expressed *AmVAchT*, *AmVGluT* and *AmGad* (Fig. 4c, e–g) and the group lateral to the ME co-expressed *AmGad* and *AmVGluT* (Fig. 4e, f, I, k, l).

**Table 4:** List of the 11 AmFoxP expressing neuron populations (clusters or groups) and their co-expressed neurotransmitter(s) (Glut: glutamate, Ach: acetylcholine) ordered from anterior to posterior

#	Type of AmFoxP neuron population	Localization	Name	Neurotransmitter	Figures
1	Group	Around (peri) antennal lobe	pAL	GABA, Glut, Ach	Figure 3
2	Cluster	Anteriodorsal to esophageal hole	adES	Glu	Figure 3
3	Group	Around (peri) medulla	pME	GABA, Glut, Ach	Figure 4
4	Cluster	Lateral to the lateral horn	lLH	Ach	Figure 5
5	Cluster	Anteriolateral to the lateral calyx	alLCA	GABA	Figure 6
6	Group	Dorsal to the central complex	dCX	Glu, GABA	Figure 8
7	Cluster	Ventral to the medial calyx	vMCA	Ach	Figure 8
8	cluster	Posteriolateral to the lateral calyx	plLCA	Ach	Figure 10
9.1	Cluster	Medioventral to the lobula, anterior	mvLO-a	Ach	Figure 10
9.2	Cluster	Medioventral to the lobula, posterior	mvLO-p	Ach	Figure 10
10	Cluster	Middle Kenyon cells	mKC	Ach	Figure 12
11	Group	Ventral to the gnathal ganglia	vGNG	GABA, Glut, Ach	Figure 7

#### GABA-ergic AmFoxP neurons

Our stainings confirmed the *AmGad* expression pattern shown by Kiya and Kubo [58]. We also found GABAergic neurons particularly in the cortical regions of the AL, the OL, the CX, but not in the KC of the MB, which is also corroborated by our RT-qPCR data (Fig. 14m). *AmGad* was detected in large clusters lateral to the AL (Fig. 3g, h, o), most likely inhibitory PNs (iPNs) [94, 101] or inhibitory local interneurons [50, 57]. The A3-v neuron cluster of the PCT [58, 95, 96] was also labeled (not shown). Only one AmFoxP cluster expressed *AmGad* exclusively, the alLCA (Fig. 6). *AmGad* expressing AmFoxP neurons were also observed in all four AmFoxP groups; around the AL (pAL, Fig. 3e, g, h, l, o), around the ME (pME, Fig. 4e, I, k), ventral to the GNG (vGNG, Fig. 7e, h, j) and dorsal to the CX (dCX, Fig. 8e, k, i). Within the pME, GABAergic AmFoxP neurons accumulated medioventral to the ME (Fig. 4e, i, k).

#### Glutamatergic AmFoxP neurons

*AmVGluT* was strongly expressed in large somata lateral to the GNG (Fig. 2c), most likely motor neurons as described previously [52], as well as in cortical areas around the medulla (Fig. 2a, Fig. 4f, l) and in clusters dorsal to the AL (Fig. 3f, n, m). Within the pAL glutamatergic AmFoxP neurons were detected anterio-mediodorsal (Fig. 3n) and posterioventral to the AL (Fig. 3i). In seven samples (Fig. 14f–j) there was also very weak *AmVGluT* signal in the small and middle KC (sKC and mKC) of the MB. In the other eight *AmVGluT* samples the KC signal did not differ from background. The adES cluster was the only AmFoxP cluster that exclusively co-expressed *AmVGlut* (Fig. 3f, m). Only few *AmVGluT* neurons were detected in the vGNG (Fig. 7f) and dorsal to the central complex (dCX, Fig. 8h). *AmVGluT*-expressing AmFoxP neurons in the dCX were located medial to *AmGad*-expressing ones. The *AmVGlut* neurons in the pAL, pME and vGNG were intermingled with AmFoxP neurons expressing *AmGad* and *AmVAchT.*

#### Monoaminergic AmFoxP neurons

The monoaminergic marker VMAT labels neurons that express histamine (HA), octopamine (OA), dopamine (DA) or serotonin (5-HT). Large clusters were located in the region of the antennal commissure (Fig. 3e, i) and ventral to the lateral calyx (LCA) (Figs. 5d, 11b), likely corresponding to the dopaminergic C1 and C3 clusters previously described [51, 102, 103]. Further large *AmVMAT* positive cells were localized in the somatic region ventral to the GNG, probably corresponding to the octopaminergic VUMmx neurons described previously [53, 83, 103]. Only a few isolated AmFoxP neurons co-localized with *AmVMAT*. They did not belong to any of the above described neuron populations (Fig. 1; Table 3). Because there were only individual neurons co-labeled by *AmFoxP* and *AmVMAT* we decided to name them ‘MF neurons’; monoaminergic AmFoxP neurons. They were detected in four regions: dorsolateral to the AL (‘MF1’), anteriomedial to the medulla (‘MF2’), dorsomedial to the lobula (‘MF3) and lateral to the upper division of the central body (CBU), part of the CX (‘MF4’) (Figs. 4d, 11).

### AmFoxP neuron populations and co-expressed neurotransmitters were consistently detected in all sampled brains

We qualitatively compared brains from individuals aged 1–50 days after emergence from the differently treated groups (Table 1). All 11 AmFoxP neuron populations were consistently detected, independent of age or treatment. The type of neurotransmitter being co-expressed was also stable and did not change (Figs. 3, 4, 5, 6, 7, 8, 9, 10).

### Monoaminergic receptors in the KC

To further refine the identity of the AmFoxP-expressing mKC [32] we checked for co-expression of the D2-like dopamine receptor AmDop3 that was shown to be restricted to the lKC [150]. While the AmDop3 sense-probe (control) did not show any unspecific staining (Fig. 12f), our *dISH* stainings with the AmDop3 antisense probe revealed no coincidence with AmFoxP label within the same neurons, but juxtaposed populations of neurons expressing AmFoxP or AmDop3 (Fig. 12g).

Figure 13 schematically summarizes the neurotransmitter profiles of AmFoxP neuron populations in the honeybee brain described above.

### *AmVAchT* and *AmVGluT* expression pattern in the KC varied in bee samples of different age

Our stainings consistently revealed AmVAchT-expression in the class I KC across all samples. This was confirmed by RT-qPCR data that revealed ten times more AmVAchT than AmVGluT mRNA levels in extracts prepared from calyces (Fig. 14m). AmVMAT and AmVIAAT levels were very low compared to AmVAchT (AmVMAT: 100 times less, AmVIAAT: 70 times less).

Interestingly, in younger bees (age 1–4) AmVAchT expression was restricted to the small and middle KC (sKC and mKC) (Figs. 2, 14a, b) whereas the large KC (lKC) only started expressing AmVAchT between the ages 11–15 (Fig. 14c, d). In very old bees (aged 40 and 50 days) the intensity of AmVAchT-expression in the sKC decreased but did no longer differ between the different types of class I KCs (Fig. 14e). Although very low in general and only visible with increased brightness and contrast (FIJI), AmVGluT expression levels in the small and medial KC were comparatively higher between 1 and 19 days after emergence than in older bees (Fig. 14f–j). Low AmVGluT levels in the KC were also detected with RT-qPCR and 7–11 times higher than AmVIAAT and AmVMAT levels (Fig. 14m).

## Discussion

The transcription factors FOXP1, FOXP2 and FOXP4 are relevant for human cognition including language [104]. They are strongly conserved in vertebrates where they play a role in sensory processing, motor behaviors and learning [105–110]. The genomes of other animals harbor only a single *FoxP* gene locus encoding a gene with a highly conserved DNA-binding domain [25, 33, 111, 112]. The fact that the neural expression pattern in vertebrates and the Forkhead box sequence in bilateria are so highly conserved raises the question whether FoxP might bestow neurons with particular properties and functions that might have been conserved since the last common ancestor of the bilateria. This type of ‘deep homology’ has been noted for a number of other transcription factors [90, 91, 113–116]. Alternatively, honeybee FoxP (AmFoxP) might have very different functions than vertebrate FoxPs, depending on the evolution of the *FoxP* gene’s regulatory sequences and differences in binding of the FoxP proteins to their target genes and other proteins [117].

As a first step towards the evaluation of these hypotheses, we used bee brains to map the distribution of isoform-specific AmFoxP expressing neurons and for some of them their projection pattern [25]. AmFoxP is expressed in particular sets of neurons in most cortical regions of the honeybee brain, many of them densely clustered, whereas others are found in a more distributed fashion [25].

In the present report we classified AmFoxP neurons by their neurotransmitters without discriminating between the two previously described AmFoxP isoforms. We examined individuals of different age and treatments to qualitatively assess whether the number of populations was rather robust or susceptible to different sensory and motor experience. FoxP2 can be regulated developmentally as well as by motor activity and sensory stimuli [13, 14, 47–49]. Overall, we found that AmFoxP neurons in the honeybee co-expressed a variety of neurotransmitters, similar to what has been reported in vertebrates. Below we will discuss the transmitter profile of different neurons in honeybee and other insect brains based on the markers we used, and propose how these neurons might relate to already described neurons and their function.

### Neurotransmitters

Neurotransmitters are essential for the identity and function of neurons. In honeybees their expression levels have been correlated to certain behaviors, e.g. novelty-seeking [118] or learning and memory [119]. Some of the proteins involved in neurotransmitter release are conserved and date back to the last common ancestor of eukaryotes [120, 121]. We focused on the most abundant neurotransmitters in the insect brain, i.e. glutamate, GABA, acetylcholine and monoamines. Using double-label in situ hybridization we revealed differential expression of their markers in AmFoxP neuron populations. The seven previously defined AmFoxP neuron clusters (adES, lLH, alLCA, vMCA, mKC, plLCA, mvLO) co-expressed only one of the four neurotransmitter markers whereas the four groups (pAL, pME, dCX, vGNG) showed a rather heterogeneous neurotransmitter profile. This finding corroborates the differentiation between the two AmFoxP neuron population classes, i.e. ‘groups’ and ‘clusters’. Within the ‘groups’ there might be smaller subunits that project to the same areas and co-express only one neurotransmitter. The pAL could certainly be further subdivided into smaller clusters lateral, medial, ventral and dorsal to the AL, being glutamatergic, GABAergic or cholinergic.

#### Acetylcholine

Three of the four AmFoxP groups (pAL, pME, vGNG) and five of the seven clusters (lLH, mvLO, plLCA, vMCA, mKC) expressed *AmVAchT*. The largest cluster, the mvLO, consists of two divisions, an anterior (mvLO-a) and a posterior (mvLO-p) part that project to different areas of the brain [25]. The neurites of the mvLO-p somata converge onto a tract that connects the lobula with the posteriolateral protocerebrum (PLP). Such a posterior cholinergic tract was previously seen with AChE histochemistry [56]. Whether the GABAergic, AmFoxP-negative neurons between the two subdivisions of the cluster converge onto the same tracts as the mvLO-a or mvLO-p requires further studies. Schäfer and Bicker [50] showed a GABA-ir tract connecting the posterior part of the lobula and the protocerebrum that could be intermingled with the AchE-tract. If this were the case the PLP would receive inhibitory and excitatory input from the lobula. The circuitry involved might be thus important to extract oscillating, like temporal features of sensory signals [122–124].

The cholinergic vMCA is reminiscent of a cluster with AchE activity ventral to the medial calyx and dorsal to the anterior optic tract (AOT) described by Kreissl and Bicker [56]. This cluster connects to the fan-shaped body/CBU, a major subdivision of the central complex which is important for polarized vision, motor control and spatial memory [125, 126]. In future studies, neuronal tracing will help to further identify the other two cholinergic AmFoxP clusters; i.e. the lLH and the plLCA.

Interestingly, *AmVachT* was highly expressed in the KC, as shown by both RT-qPCR and *dISH*. Staining was punctate and coincided less with cell bodies, in contrast to the *AmVachT* staining observed in the rest of the brain. Because we do not assume local translation and the puncta were numerous we do not think that these signals are located synapses contacting the KC somata which were described only for dopaminergic synapses [127]. Due to the concentrated and punctuate staining pattern it is difficult to determine whether *AmFoxP* and *AmVachT* co-localized in all mKC. Based on our analysis (see “Cholinergic AmFoxP neurons” section) we conclude that they predominantly do. Until recently there was no strong evidence about the identity of KC neurotransmitters, one of the main limitations being the difficulty to isolate postsynaptic neurons to KC to characterize physiologically their neurotransmitter profiles. Previous studies in the honeybee described the expression of *Acetylcholinesterase* (*AchE)* in KC [56, 128]. Shapira et al. [128] showed expression in all class I KC in nurses, but restricted signal in the lKC in foragers. *AmVachT* expression in our study was restricted to the sKC and mKC in young bees and expanded to all class I KC in older bees. These differences could be explained by the different biosynthetic origins of the AchE enzyme and the vesicular transporter VAchT. AchE is associated to the neurotransmitter cycle by degrading acetylcholine in the synaptic cleft. In vertebrate neuromuscular junctions it can be released pre- and post-synaptically [129] which makes it a marker for cholinergic as well as cholireceptive neurons [130]. Also, it is implicated in other metabolic pathways [131, 132]. Thus AchE produced by the KC might be predominantly transported to the microglomeruli in the calyx neuropil to hydrolyze Ach transmitted by projection neurons. Barnstedt et al. [133] demonstrated that some KC are cholinergic in *Drosophila* and our finding constitute the first unequivocal evidence that KC are cholinergic in the honeybee.

#### Glutamate

All four AmFoxP groups and one cluster, the adES, expressed *AmVGluT*. In VGlut-reporter *Drosophila* lines, several large glutamatergic neurons with a similar distribution as the neurons of the honeybee dCX cluster were shown to project into the central complex (CX) [134]. Thus, the AmFoxP neurons of the dCX might be a subset of glutamatergic neurons also projecting into the CX. This proposition is supported by the study on *Drosophila dFoxP* gene activity in the CX by Lawton et al. [27]. As the CX neuropils strongly express GluCl receptors [64], the glutamatergic AmFoxP neurons of the dCX might transmit inhibitory input. Based on location, the glutamatergic AmFoxP neurons in the vGNG cluster might be descending (motor) neurons, possibly innervating neck muscles [135, 136].

In some sections, especially in newly emerged bees, sparse and very weak signals of *AmVGluT* were detected in the sKC. This was corroborated by the RT-qPCR data showing that the KC expressed small amounts of *AmVGluT*, ten times less than *AmVAchT*, but 7–11 times more than *AmVIAAT* and *AmVMAT*. The very low levels of the latter two markers might be due to contamination of the samples with cells other than KC. Very weak glu-ir signals [52] and high mRNA amounts of *AmEAAT* [59] in the sKC of the MB have been described before [52, 59]. In crickets, glu-like-ir signal was reported only in the (inner) KII and (outer) K III but not in the (inner) KI KC [54]. In *Drosophila*, glu-like-ir was detected in the αβ_c_—KC subset, which is developmentally generated last and expresses dFoxP [28, 32], but not dVGluT [55]. These authors discuss that neurotransmitter expression in the KC could vary over lifetime and that glutamate might be expressed only transiently. This is in line with our data, as we see weak signals in younger bees at the age of 1–19 days, but less distinctive or not at all in older bees, aged 23–50 days. This age-dependent difference could mirror the behavioral switch from nurses to foragers which is accompanied by gene expression differences [137].

#### GABA

The four AmFoxP groups and the alLCA cluster express *AmGad*. AmFoxP neurons dorsal to the CX (dCX) that co-express *AmGad* might correspond to neurons described by GABA-like-ir [50] and project to the CX. If the glutamatergic AmFoxP neurons connected to postsynaptic neurons with GluCl receptors in the CX, as mentioned above, then the dCX cluster would transmit only inhibitory input to the CX. Strausfeld and Hirth [91] have compared the CX to the vertebrate basal ganglia, based on similar developmental gene expression profiles, similar function in the selection and maintenance of adaptive behavior and because both constitute a ‘midline brain structure’ [91]. Interestingly the striatum of the basal ganglia express FoxP1, 2 and 4 [14, 21, 23, 138–140]; and plays a role in adaptive sensorimotor behavior [141–144]. Similarly, the CX is important for sensorimotor integration and motor control [91, 145–147] and it will be interesting for future studies to examine a possible corresponding role of the inhibitory AmFoxP neurons that might project to the CX.

The single *AmGad*-expressing AmFoxP neurons in the pAL neuron group are located anterio-dorsal and posterio-ventral to the AL but not in the larger *AmGad*-labeled clusters lateral to the AL which are most likely inhibitory projection neurons [94, 101]. The GABAergic AmFoxP neurons within the pAL might be local interneurons [57, 148]. We also showed that the AmFoxP neurons in the mvLO are not GABAergic and thus confirmed that they do not correspond to the (more anteriorly located) A3v cluster as suggested by Kiya et al. [31].

#### Monoamines

We found overlap between *AmVMAT* and *AmFoxP* expression only in less than ten isolated *AmVMAT*-expressing AmFoxP neurons throughout the brain whereas none of the eleven described AmFoxP neuron populations expressed *AmVMAT*. The isolated *AmVMAT*-expressing neurons around the mvLO might be part of the S_L_ cluster that projects dorsally [51, 102]. The MF1 might correspond to the 2–3 dopaminergic soma between the AOTU and the AL described by Tedjakumala et al. [102]. Because of their localization rather dorso-lateral to the lobula, the MF3 neurons could not be part of the dopaminergic C4 cluster [102]. Mercer et al. [103] also located a catecholamine-labeled neuron cluster dorso-medial to the lobula, as did Nässel et al. [149] for serotonin. The MF4 might belong to the ‘S_P_-cluster’ that projects into the central body and the noduli [51].

We also used *AmDop3* to detect the dopaminergic D2-like receptor, previously reported to be expressed in the lKC [150]. There were only individual—DAPI identified—KC nuclei being labeled by both, the *AmFoxP* and the *AmDop3* probes which supports the suggestion by Suenami et al. [151] that AmFoxP expressing KC could belong to the lKC. However, mostly, *AmDop3* and *AmFoxP* did not overlap. This is consistent with the idea that there is a separation between different KC subgroups, but it is not always sharp.

We did detect monoamines also in the previously described dopaminergic clusters C1, C3 and the S-clusters, which we found to be strongly labeled by *AmVMAT*, whereas C2 and C4 were not labeled. This could be due to differential sensitivity of the markers used. The previous studies detected immunoreactivity for Tyrosine Hydroxylase (TH) the enzyme that catalyzes l-tyrosine to L-DOPA, the precursor for dopamine [51] and immunoreactivity for GABA [102]. Furthermore, our staining labeled the large and strongly expressing octopaminergic VUMmx neurons ventral to the GNG [53]. We unambiguously identified the VUMmx neurons by their projection pattern using neuronal backfilling [53, 83]. We also observed some very weakly-labeled *AmVMAT* neurons close to the AL that might express serotonin as described in Dacks et al. [152].

In vertebrates, FoxP2 is expressed in the ventral tegmental area and substantia nigra [20, 23] which are the main sources of striatal and limbic forebrain dopamine. However, explicit cellular co-localization of dopamine and FoxP2 has not been reported for these regions.

#### Neurotransmitter profile of vertebrate FoxP neurons

Some developmentally relevant transcription factors are only expressed in neurons of a particular neurotransmitter type [153, 154]. This is not the case in our study of honeybees and also not in vertebrates. For instance, FoxP2 and GABA co-occur in the Purkinje cells, striatal medium spiny neurons [14, 20], ‘arkypallidal’ neurons of the external Globus pallidus [155], neurons ventromedial to the ‘Barrington’ nucleus (‘Bar’, pons, brain stem) [156] and in the parabrachial nucleus (‘PB’, pons, brain stem) [157]. FoxP2 and glutamate co-occur in pyramidal neurons in the cortex [20], the dLGN (thalamus) [46], neurons dorsolateral to the Bar nucleus [156], in the subparabrachial nucleus [158] and in the PB (pons, brain stem) [157]. Cholinergic spinal motor neurons express FoxP1, FoxP2 and FoxP4 during neuronal differentiation [159–162]. FoxP2-expressing neurons in the substantia nigra [20] are likely to be dopaminergic. Which of these neuron populations might have similarities (beyond FoxP and a specific neurotransmitter profile) to neurons in honeybee, requires more comparative information on molecular profiles of particular AmFoxP expressing neurons, their anatomical connections and their function.

### Aging/environmental stimulus manipulation

Age and environmental factors determine many aspects of life in honeybees. For this reason, we investigated whether age or environmental conditions play a role in the co-expression of neurotransmitters and AmFoxP in the analyzed neuron populations. After emerging from their cells, honeybee workers stay in the hive for about 3 weeks and perform tasks sequentially, i.e. cell cleaning, nursing, wax production and guarding. Subsequently, they start to forage for water, nectar or pollen and communicate their findings with the highly sophisticated ‘waggle dance’ behavior to their sisters in the hive [38–41, 163]. They die about 6 weeks after emersion. Honeybee brain anatomy [164] and the expression levels of certain proteins like CREB [165], bruchpilot [166] vitellogenin [167], synapsin [168] or neuropeptides [169] vary with age and experience. Seasonal changes in gene expression were also observed [170–172]. FoxP2 levels also change in a specific brain area of songbirds with age [14] and as a result of singing [47–49]. For this reason, we hypothesized that some AmFoxP clusters and groups might be dynamic in terms of *AmFoxP* and neurotransmitter expression. However, we could identify the clusters and groups based on FoxP expression reliably in all brain samples analyzed, regardless of age and environmental condition. While this finding is not quantitative it suggests that under the housing and seasonal conditions we used *AmFoxP* and the associated neurotransmitters did not vary noticeably. We conclude that the neuron populations, we identified, are not functionally restricted to certain life-cycle phases, but instead needed for behavior that might be constantly relevant. Using RT-qPCR Kiya et al. [31] detected an age-dependent increase of *AmFoxP* expression in whole-brain samples from eclosion to age 34 days but no significant differences between nurse and worker bees. We also found an increase of *AmFoxP* transcript from pupae to workers aged 33 days [25]. We conclude that the results of Kiya et al. [31] and ours reflect a different sensitivity than our in situ hybridization protocol affords. A combination of laser-capture and RT-qPCR could more precisely quantify the actual *AmFoxP* amounts within the eleven clusters and groups at different life history stages.

## Conclusion

In summary we showed that each of the seven AmFoxP neuron *clusters* co-expressed only one particular neurotransmitter whereas the four *groups* co-expressed more than one neurotransmitter and might therefore be further sub-divided. Five clusters expressed *AmVAchT* and are therefore excitatory whereas one cluster (alLCA) co-expressed *AmGad* and is therefore inhibitory. The glutamatergic adES cluster could be inhibitory or excitatory, depending on the receptor type in the postsynapse. None of the eleven AmFoxP neuron populations co-expressed *AmVMAT*, they are therefore not modulatory. However, a small number of isolated neurons in different parts of the central brain showed co-expression of *AmFoxP* and *AmVMAT*.

All eleven AmFoxP neuron populations kept their neurotransmitter profiles across ages and under different conditions of sensory and motor deprivation. This indicates that the FoxP expressing neurons in honeybees do not undergo neurotransmitter switching [173]. Our present data provide a framework to pursue further comparative studies on the function of particular populations of invertebrate and vertebrate FoxP neurons in the context of ‘deep homology’, which can lead to insights to questions of evolutionary conservation and novelty.

## Abbreviations

See Table 5.

**Table 5:** List of anatomical and molecular abbreviations

*Anatomical abbreviations*	
AL	Antennal lobe
CX	Central complex
CBU/CBL	Central body, upper and lower division
ES	Esophageal hole
GNG	Gnathal ganglia
KC (sKC, lKC, mKC)	Small, large and middle Kenyon cells
LA	Lamina
LCA	Lateral calyx
LH	Lateral horn
LO	Lobula
MCA	Medial calyx
ME	Medulla
OL	Optic lobe
P	Protocerebrum
PB	Protocerebral bridge
PLP	Posteriolateral protocerebrum
*Am*	*Apis mellifera*
*Molecular abbreviations*	
AmVAchT	The honeybee vesicular transporter for acetylcholine
AmDop3	The honeybee D2-like dopamine receptor
AmVGluT	The honeybee vesicular transporter for glutamate
AmGad	The honeybee glutamate decarboxylase
AmVIAAT	The honeybee vesicular transporter of inhibitory amino acids
AmVMAT	The honeybee vesicular transporter for monoamines
dISH	Double-lable in situ hybridization
GABA	Gamma-aminobutyric acid
glu	Glutamate
-ir	Immunoreactive immunoreactive
